# Antisense oligonucleotide selection scheme for rare Duchenne muscular dystrophy mutations: Application to *DMD* exon 16 skipping

**DOI:** 10.1016/j.omtn.2026.103048

**Published:** 2026-08-03

**Authors:** Marine Geoffroy-Guiraud, Cyriaque Beley, Stephany Campuzano, Thu Huyen Nguyen, Luisa M. Schmidt, Christophe Blaszykowski, Marcus Krueger, Luis Garcia, Simon Guiraud

**Affiliations:** 1 SQY Therapeutics, 2 Rue Hélène Boucher, Parc Ariane, Bâtiment Pluton, 78280 Guyancourt, France; 2 Institute for Genetics, Cologne Excellence Cluster on Cellular Stress Responses in Aging-Associated Diseases, University of Cologne, 50931 Cologne, Germany; 3 Novo Nordisk Foundation Center for Protein Research, Department of Cellular and Molecular Medicine, Faculty of Health and Medical Sciences, University of Copenhagen, Copenhagen, Denmark; 4 Université de Versailles St- Quentin, Pharmacoligo UR20261, UFR des Sciences de la Santé, Montigny-le-Bretonneux, France

**Keywords:** MT: Oligonucleotides: Therapies and Applications, oligonucleotide antisense, exon skipping, high-throughput screening, screening methodology, clinical translation, Duchenne muscular dystrophy, dystrophin

## Abstract

Over the last decades, antisense oligonucleotides (AONs) have emerged as powerful tools to modulate alternative splicing, allowing exclusion of a specific exon from a target mRNA to bypass mutations or restore the reading frame, enabling production of a functional protein. In Duchenne muscular dystrophy (DMD), a lethal X-linked disorder affecting 1:5,000 males, four AON therapies have received accelerated approval. However, these treatments are applicable to only 27% of patients, leaving most individuals with rare mutations without therapeutic options. Here, we outline a methodology to identify relevant AONs for such populations. This approach aims to prequalify candidates and eliminate unsuitable leads before animal testing, reducing development time and costs. Following high-throughput screening, selected AONs are assessed for intravenous delivery compatibility and serum protein binding to support model selection and forecast human pharmacokinetics. As proof of concept, we applied this strategy to tricyclo-DNA AONs targeting an ultra-rare *DMD* exon 16 mutation. From a 59-member library, we defined a lead compound with efficient exon 16 skipping, favorable pharmacokinetic and safety profiles, able to rescue dystrophic transcriptomic and proteomic signatures. This study demonstrates the feasibility of developing a safe and effective lead compound within a reasonable time frame and cost, supporting compassionate use and future *n* = 1 trials.

## Graphical abstract

**Figure:**
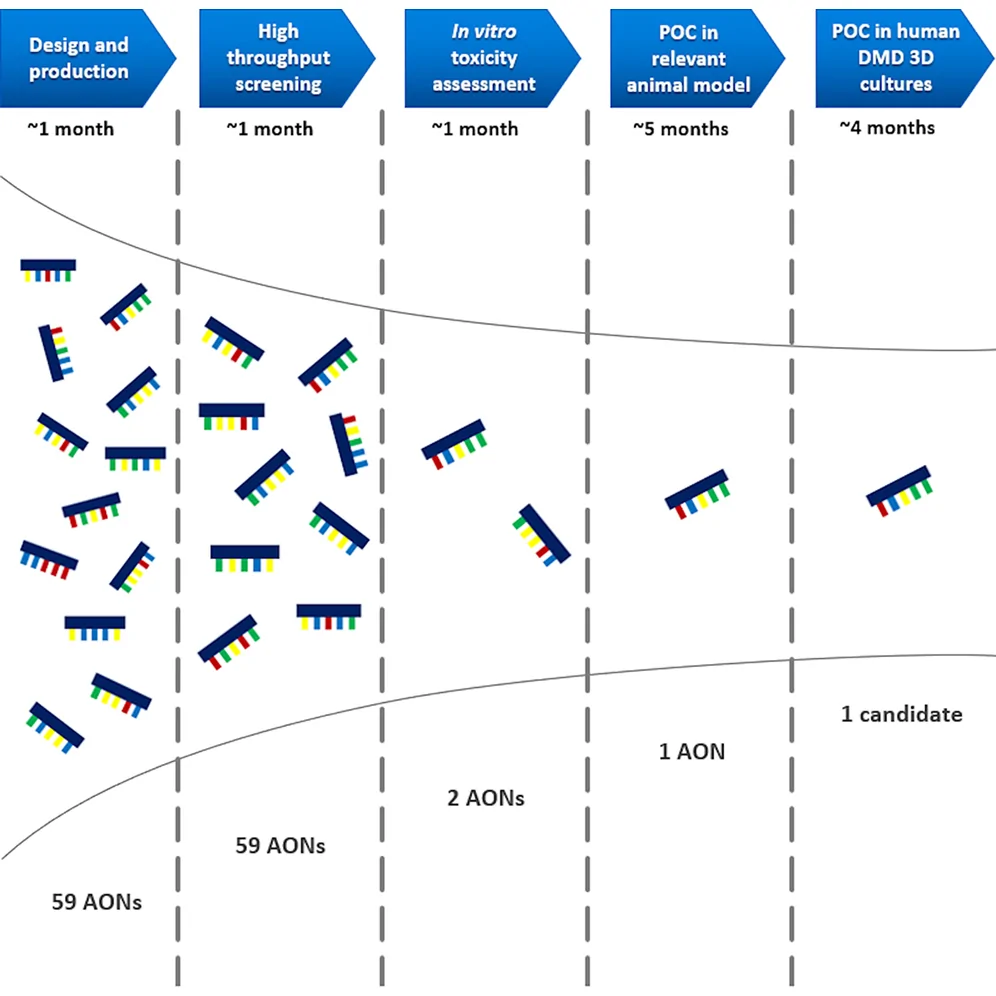

## Teaser

Guiraud-Geoffroy and colleagues present a rapid methodology to identify antisense oligonucleotides (AONs) targeting rare Duchenne muscular dystrophy mutations. Exemplified by the case of DMD exon 16 skipping, the strength of this approach lies in its ability to prequalify candidates and eliminate unsuitable leads early, before animal testing, thereby minimizing both time and costs.

## Introduction

Duchenne muscular dystrophy (DMD, MIM no. 310200) is a lethal X-linked neuromuscular disorder affecting 1 in 5,000 new-born males.^1^^,^^2^ The disease is characterized by progressive muscle degeneration, loss of walking ability by the age of 10–12 years and cardiac and respiratory insufficiencies^3^ leading to early death in the late twenties.^4^ DMD is caused by loss-of-function mutations in the *DMD* gene,^5^ mostly out-of-frame deletion disrupting the open reading frame of the dystrophin mRNA and leading to the absence of functional dystrophin protein.^6^ Dystrophin, a vital 427 kDa cytoskeletal component of the dystrophin-associated protein complex (DAPC) at the sarcolemma, connects the extracellular matrix to the actin cytoskeleton and provides structural stability to the skeletal muscle and protection to the myofibers during muscle contraction-relaxation cycles.^7^ While initially described as an essential structural unit acting as a molecular spring in skeletal muscles, accumulating evidence recently implicated dystrophin in a variety of non-mechanical and signaling roles.^8^^,^^9^^,^^10^ In the absence of dystrophin, the sarcolemma is destabilized and DMD patients experience repeated cycles of muscle necrosis and regeneration leading to eventual replacement of muscle fibers by adipose and connective tissue.^11^

Despite exhaustive clinical management of cardiac complications, assisted ventilation and corticosteroid treatment, there is currently no cure for DMD. The urgency to seek an effective disease-modifying therapy for DMD has resulted in parallel efforts to develop gene correction strategies such as exon skipping^12^^,^^13^^,^^14^ and stop codon readthrough,^15^ gene replacement,^16^ and pharmacological strategies.^17^ Exon skipping mediated by antisense oligonucleotides (AONs) aims, by interfering with the splicing machinery, to force the exclusion of a specific exon or exons to rescue dystrophin production by converting a mutated out-of-frame *DMD* transcript into a shorter in-frame mRNA,^12^ as observed in Becker muscular dystrophy (BMD, MIM no. 300376) patients.^18^ Theoretically, single or double-exon skipping approaches could restore the reading frame for 90.3% of all reported genetic defects associated with DMD, with exon 51, 45, and 53 skipping applicable to 11%, 9%, and 8% of patients, respectively.^19^ Two decades of extensive and successful preclinical studies^20^ demonstrated the great potential of exon-skipping strategies and paved the way toward clinical investigations. To date, four first-generation phosphorodiamidate based-AONs received an FDA-approval for exon 51 (eteplirsen^21^), exon 53 (golodirsen^22^ and viltolarsen^23^), and exon 45 (casimersen^24^). Despite great promises, many bottlenecks related to suboptimal distribution to target tissues and subsequent efficacy remain to be addressed. Accordingly, new backbone modifications and delivery-enhanced chemistries are under active investigation.^25^ Bioconjugation of AONs, notably with cell-penetrating peptides (CPPs)^26^^,^^27^ and anti-transferrin receptor 1 antibody^28^^,^^29^ have been shown very promising in animal models and are currently evaluated in several trials for DMD.^30^ Among delivery-enhanced chemistries, tricyclo-DNA (tcDNA)^31^ has emerged as a promising next-generation AON backbone.^32^ Seminal studies using tcDNA AONs^33^ and subsequent pre-clinical studies from SQY Therapeutics^34^^,^^35^^,^^36^ reported superior pharmacological activity over first-generation exon-skipping drugs and highlighted unique pharmacological properties as well as unprecedented systemic uptake of tcDNA-AONs in multiple DMD animal models.^33^^,^^37^^,^^38^^,^^39^^,^^40^^,^^41^^,^^42^ Biodistribution is further enhanced when the tcDNA based-AON is conjugated to a palmitoyl moiety (PALM).^35^^,^^36^

Although exon skipping for DMD has been under investigation for nearly thirty years, only a handful of AONs targeting a limited number of exons have entered in clinical trials with results that are promising so far but not yet fully conclusive. The process of identifying and advancing a lead compound to the clinic is challenging, expensive, requires many years of preclinical studies, and is often disappointing when the selected AON does not fulfill all expected prerequisites in patients. Here, we outline a methodology that allows lead compounds likely to fail at later stages of development to be rejected early. The key strength of this approach lies in its ability to prioritize the sequence of tasks: starting from the medical need (e.g., skipping a pathogenic exon to treat a specific condition) through the establishment of robust proof of concept for a final lead AON, ready for clinical development. The final AON may not necessarily be the *a priori* most effective one, but rather the one that best combines efficacy with the fewest potential liabilities. Prequalifying candidate AONs as early as possible, before animal testing, minimizes both the duration and costs of research. Following high-throughput screening of AON collections, the compatibility of selected AONs with intravenous delivery is assessed through several *in vitro* assays using blood samples from relevant species, and the serum proteins that specifically bind selected AONs are identified in blood samples from different species. This approach reduces the use of irrelevant animal species for subsequent *in vivo* studies and allows to focus on animal models exhibiting similar patterns of binding profiles, thereby improving the prediction of the pharmacokinetic profile in humans. Whenever possible, a pilot experiment in non-human primates (NHPs) is performed to assess acute toxicity and document pharmacokinetic data prior to the mandatory Good Laboratory Practice (GLP) toxicology studies required for investigational new drug (IND) and clinical trial application (CTA) submissions. In parallel, selected AONs are tested in appropriate human DMD 3D mini-muscle culture to characterize *ex vivo* pharmacokinetics and pharmacodynamics profiles in a highly differentiated dystrophic human muscle context.

To illustrate this methodology, we focused on the identification of a tcDNA compound targeting an extremely rare *DMD* exon 16 mutation. Only a few studies have previously highlighted that loss of exon 16 does not impair dystrophin function in human^43^ and have demonstrated the ability of AONs to efficiently skip *DMD* exon 16.^44^ The choice of exon 16 as an example to illustrate this methodology is particularly relevant because the DMD population eligible to exon 16 skipping is extremely small, and consequently this target is currently not being pursued in commercially driven clinical development programs, despite the fact that treatment would be feasible. Whereas the absence of animal models for rare mutations—such as exon 16 deletion—hampers conventional preclinical testing, the added value of selecting *DMD* exon 16 was to demonstrate the ability to obtain a lead compound within a reasonable time frame.

## Results

### Design, screening, and *in vitro* selection of tcDNA candidates to efficiently skip exon 16

**Figure 1:**
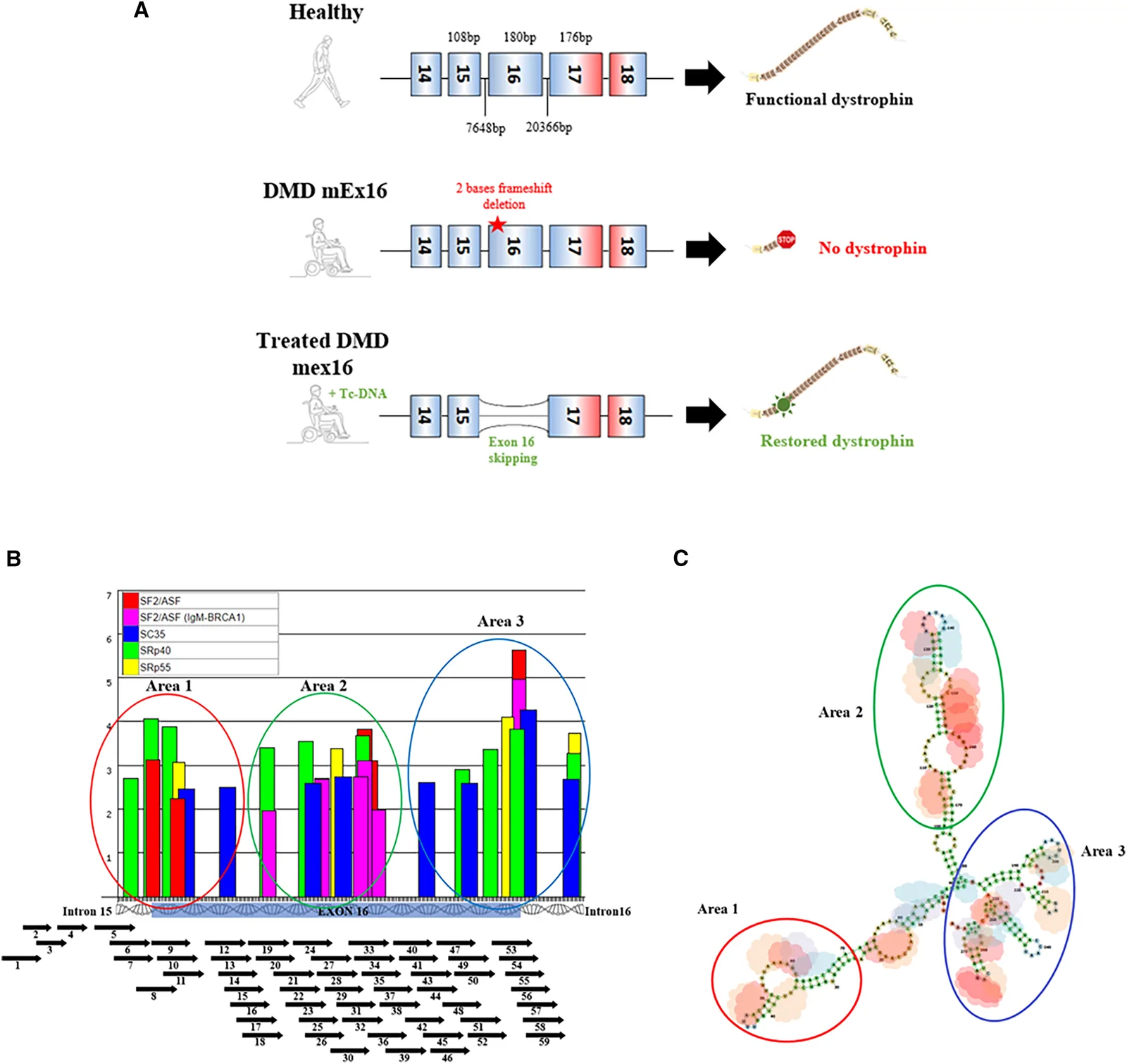
AONs design for the discovery of tcDNA antisense oligonucleotides targeting exon 16 of the human dystrophin gene (A) Schematic representation of the exon 16 skipping strategy in *DMD* pre-mRNA. (B) Predicted exonic splicing enhancer (ESE) elements by ESE Finder tool within the intron 15-exon 16-intron 16 region of *dystrophin* mRNA, showing the targeted region and localization of the 59 designed tcDNA sequences. (C) Predicted three-dimensional structure of *DMD* exon 16 using RNAFold tool. The RNA sequence of the *DMD* exon 16 (180nt) with 50 nucleotides on the introns 15 and intron 16 for a 280nt sequence in total was used. The colored clouds indicate the localization of the different ESEs (SF2/ASF, SF2/ASE (IgM-BRCA1), SC35, SRp40, and SRp55) presented in Figure 1B.

To illustrate the proposed discovery cascade for AON-mediated exon skipping, we used the immortalized human DMD myoblast line (hDMD mEx16) derived from a quadriceps biopsy of a 16-year-old DMD patient suffering from a rare two-nucleotide deletion in exon 16. This deletion disrupts the dystrophin mRNA reading frame, generates a premature translation stop in exon 17 (Figure S1), and consequently abolishes dystrophin protein production, resulting in the DMD phenotype. Skipping of exon 16 has the therapeutic potential to restore the reading frame, re-establish dystrophin production, and thereby counteract the disease phenotype (Figure 1A).

Dystrophin protein is not expressed at the myoblast stage,^45^ and to ensure proper protein comparison at the myotube level, we first evaluated by quantitative reverse-transcription PCR (RT-qPCR) the myogenic potential of hDMD mEx16 cells relative to healthy control. The expression of myoblast (Myf5), myocyte (MyoD), and myotube markers (MRF4, myogenin, desmin, and titin) demonstrated comparable myogenic regulatory patterns between both cell lines (Figure S2).

**Figure 2:**
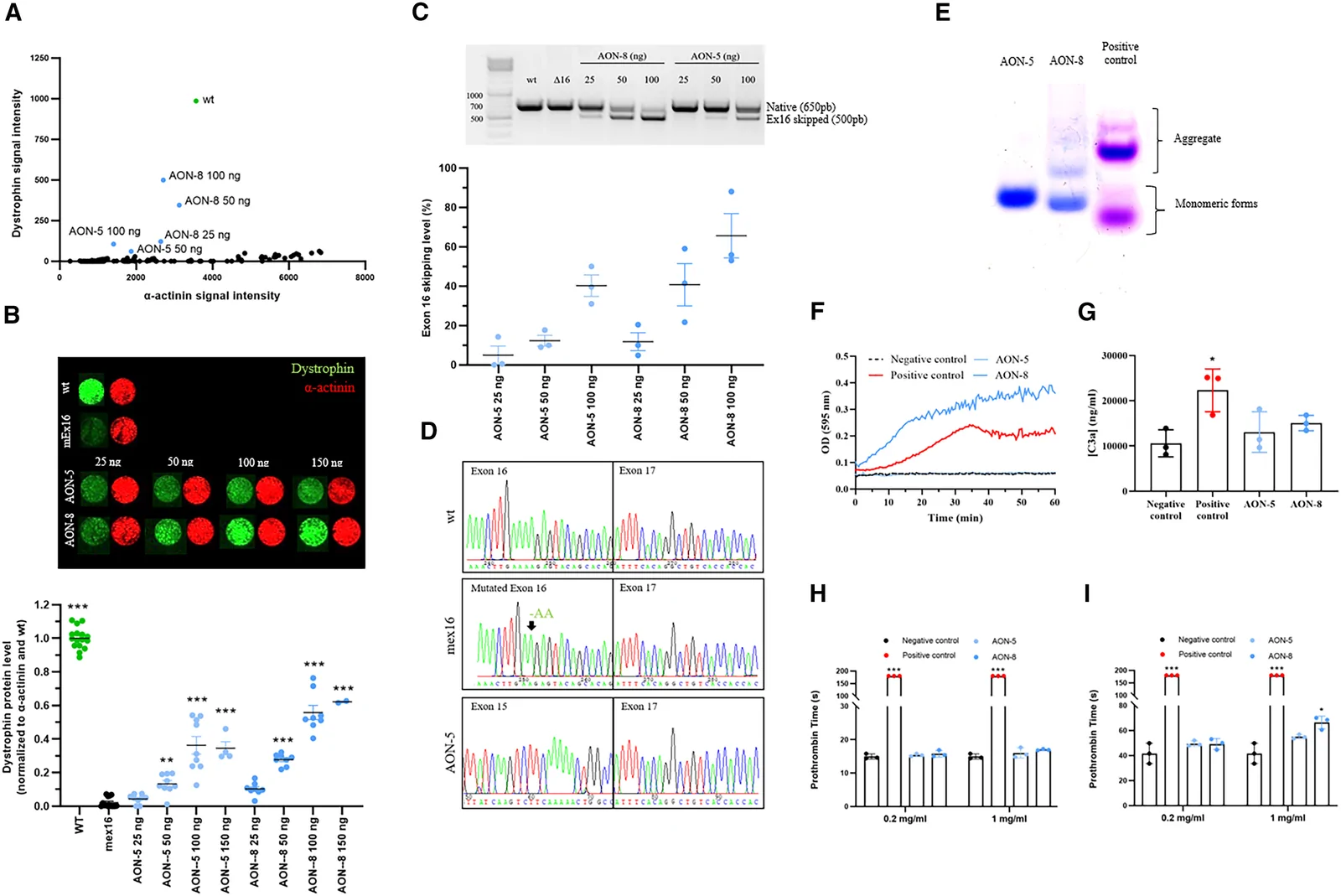
High-throughput screening, hit validation, and toxicity assessment of exon 16-skipping tcDNA (A) Dystrophin protein levels following treatment of hDMD mEx16 myotubes with tcDNA designed to skip *DMD* exon 16. (B) Quantification of dystrophin expression in hDMD mEx16 myotubes treated with increasing concentrations of AON-5 or AON-8 (25, 50, 100, and 150 ng). *p* < 0.05∗, *p* < 0.01∗∗, *p* < 0.001∗∗∗; analyzed by one-way ANOVA. Results are expressed as the mean ± SD (*n* = 16 for WT and mEx16; *n* = 9 for 25, 50, and 100 ng AON-5 and AON-8 treated groups; *n* = 4 for 150 ng AON-5- and AON-8-treated groups). (C) RT-PCR amplification of exons 15–18 in wild-type, hDMD mEx16, and AON-5- or AON-8-treated hDMD mEx16 myoblasts showing a dose-dependent exon 16 skipping effect. (D) Sequencing chromatograms from wild-type, untreated, and AON-5-treated hDMD mEx16 myoblasts confirming exon 16 exclusion. (E) Tris-acetate gel electrophoresis analysis of AON-5 and AON-8 to assess potential homodimer formation. (F) Flocculation assay of AON-5 and AON-8 in human plasma. (G) Complement activation assay measuring C3a generation following incubation of AON-5 and AON-8 in human serum. (H and I) (H) Prothrombin time (PT) and (I) activated partial thromboplastin time (aPTT) assays evaluating the effects of tc-5 and tc-8 on coagulation in human plasma. (*p* < 0.05 (∗), *p* < 0.01 (∗∗), *p* < 0.001 (∗∗∗); analyzed by one- or two-way ANOVA. Results are expressed as the mean ± SD (*n* = 3 per groups).

We next designed a comprehensive PALM-tcDNA “walk” consisting of 59 full phosphorothioate-modified and 5' palmitoyl-conjugated 15- and 20-mer tcDNA AONs targeting exon 16 and its adjacent intronic regions. Design parameters included nucleotide count, GC content, and melting temperature (Table S1). *In silico* analyses of predicted exonic splicing enhancers (ESEs) using ESE Finder tool^46^ as well as mRNA secondary conformation (RNA fold tool^47^) highlight 3 areas of interest to orientate tcDNA design and correlate their efficacies to accessible mRNA regions enriched in ESEs (Figures 1B and 1C). All designed PALM-tcDNAs were subjected to an initial high-throughput screening assay to assess their ability to restore dystrophin protein expression following PALM-tcDNA treatment. PALM-tcDNAs were transfected into hDMD mEx16 myoblasts at doses of 25, 50, and 100 ng/100 μL, and dystrophin expression was quantified by In-Cell Western (ICW) assay^48^ 96 h post-treatment and following myotube differentiation. As illustrated in Figure 2A, treatment with AON-5 and AON-8 induced a dose-dependent restoration of dystrophin protein expression, up to 50% of wild-type levels. Independent replicates confirmed these findings, with AON-5 achieving 4%, 13%, 36%, and 35%, and AON-8 achieving 10%, 28%, 56%, and 62% restoration of dystrophin protein at 25, 50, 100, and 150 ng, respectively (Figure 2B). Consistently, exon-skipping analysis by reverse-transcription PCR (RT-PCR) revealed 5%, 12%, and 40% (AON-5) and 12%, 41%, and 66% (AON-8) exon 16 skipping efficiencies (Figure 2C), with sequencing confirming exon 16 exclusion after tcDNA treatment (Figure 2D). Interestingly, AON-5 and AON-8 localized at the intron 15-exon 16 junction, in the open and ESEs-rich area 1 (Figures 1B and 1C), and these results are in line with previous work from Wilton’s lab which achieves *DMD* exon 16 skipping targeting this same area using 25–31 nt AONs.^44^

Following identification of the two most potent 20-mer PALM-tcDNA sequences promoting dystrophin restoration in patient-derived cells, we conducted *in vitro* safety evaluations to confirm their suitability as candidate compounds. As previously reported, AONs may exhibit sequence-dependent toxicity due to homodimer formation, potentially leading to complement activation, prolonged coagulation, or platelet activation.^36^ Consistent with *in silico* predictions (Table S1), AON-8 formed dimers and precipitated in human plasma, whereas AON-5 displayed a favorable safety profile (Figures 2E and 2F). Complement activation assays in human serum, as well as prothrombin time (PT) and activated partial thromboplastin time (APTT) assays, revealed no complement activation for either compound (Figure 2G). However, AON-8 induced a significant prolongation of clotting time in APTT assays, suggesting an anticoagulant effect and a potential risk of excessive bleeding (Figure 2I). In contrast, AON-5 did not alter coagulation parameters in human (Figures 2H and 2I) and monkey plasmas (Figure S3). Although further sequence refinements (e.g., wobble pairing) could mitigate AON-8 toxicity by preventing homodimerization,^36^ AON-5 was selected as the lead candidate based on its robust *in vitro* efficacy and favorable non-clinical safety profile.

Taken together, these *in vitro* screening studies using human DMD cells led to the identification and selection of AON-5, henceforth referred as PALM-SQY16, the most efficient and safe AON candidate to induce exon 16 skipping and restore dystrophin protein expression. Overall, these results define a robust and fast-moving *in vitro* cascade for selecting potent PALM-tcDNA candidates for subsequent preclinical development.

### Proof-of-concept study in wild-type mice

**Figure 3:**
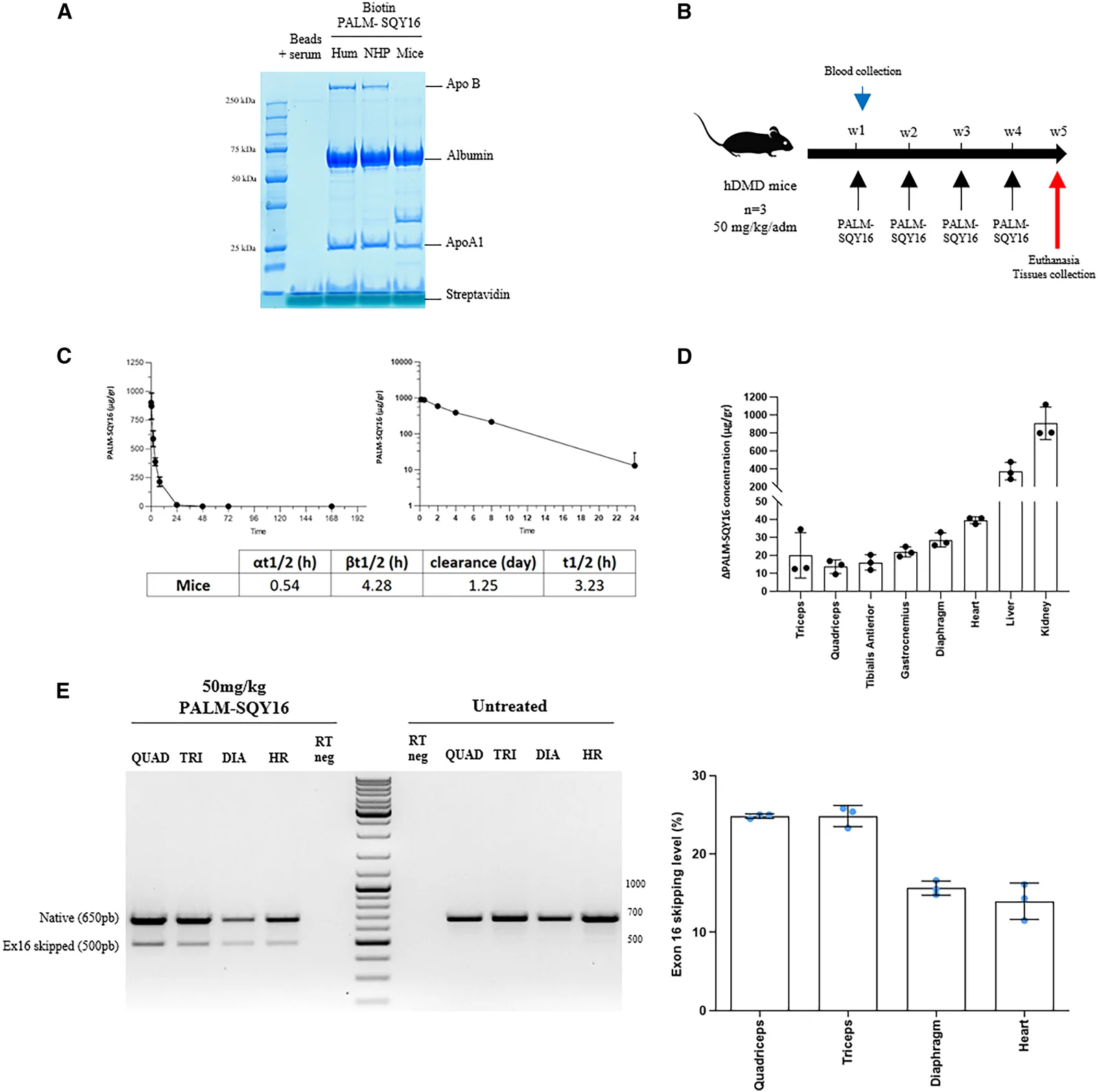
Proof-of-concept study of PALM-SQY16 in hDMD mice (A) Protein binding profile of biotinylated PALM-SQY16 revealed by high-capacity streptavidin agarose pull-down assay. (B) Schematic representation of the 4-week pharmacokinetic study design in hDMD mice treated with PALM-SQY16 at 50 mg/kg/week. (C) Serum concentration-time profile of PALM-SQY16 in hDMD mice following administration of a 50 mg/kg dose. (D) Quantification of the metabolite ΔPALM-SQY16 in skeletal, respiratory, and cardiac muscles, as well as in liver and kidney, after 4 weeks of treatment with PALM-SQY16 (50 mg/kg/week). (E) Detection of exon 16-skipped *dystrophin* mRNA (left) and quantification of exon 16 skipping levels (right) in skeletal, respiratory, and cardiac muscles after 4 weeks of PALM-SQY16 treatment (50 mg/kg/week). Data are presented as mean ± SD; *n* = 3 mice per group.

The enhanced potency of palmitic acid-conjugated tcDNAs has previously been attributed to their improved binding affinity with serum albumin.^35^^,^^49^ Therefore, prior to *in vivo* evaluation, we performed proteomic profiling of PALM-SQY16 in human, murine, and monkey sera. In all tested species, PALM-SQY16 primarily interacted with albumin and apolipoprotein A1 (Figure 3A; Table S2). Notably, PALM-SQY16 binding to albumin was comparable between human and monkey sera, and approximately 2 ± 0.5-fold higher than in mouse serum (Table S2).

Given the lack of a suitable DMD animal model to assess exon 16 skipping, we investigated the pharmacokinetic (PK) and pharmacodynamic (PD) profiles of PALM-SQY16 in hDMD mice—C57BL/6J transgenic animals carrying an intact and functional copy of the 2.3 Mb human dystrophin gene (h*DMD*) stably integrated into murine chromosome 5.^50^ This model has proven valuable for the preclinical assessment of human sequence-specific therapeutics.^51^ Mice received weekly intravenous injections of PALM-SQY16 (50 mg/kg) for four weeks (Figure 3B). The resulting PK profile revealed an estimated 3.23 h terminal half-life and a clearance time of 1.25 days (Figure 3C). Following tissue uptake, PALM-SQY16 undergoes de-palmitoylation to generate the main metabolite ΔPALM-SQY16, which persists in tissues but is no longer detectable in blood. To evaluate tissue distribution, we therefore quantified both palmitoylated (PALM-SQY16) and deconjugated (ΔPALM-SQY16) forms in skeletal, respiratory, and cardiac muscles, as well as in the kidney and liver—organs known to accumulate AONs.^35^^,^^36^ As shown in Figure 3D, only the ΔPALM-SQY16 form was detected in tissues. Concentrations ranged from 14 ± 2.8 to 40 ± 1.4 μg/g in quadriceps, triceps, diaphragm, and heart, while higher levels were observed in the liver (373 ± 69.9 μg/g) and kidney (905 ± 139.7 μg/g). This biodistribution profile correlated with exon 16 skipping efficiencies of 25% ± 0.2% in quadriceps, 25% ± 1.0% in triceps, 16% ± 0.6% in diaphragm, and 14% ± 1.6% in heart (Figure 3E). These results are consistent with previously published data obtained using similar tcDNA chemistries and dosing regimens targeting exon 23 in *mdx* mice.^35^

Overall, these results establish the PK/PD profiles of PALM-SQY16 in mice and demonstrate efficient biodistribution and exon 16 skipping activity in skeletal, respiratory, and cardiac muscles, confirming the suitability of PALM-SQY16 for further preclinical evaluation.

### Efficacy, PK/PD, and preliminary safety studies in NHPs

**Figure 4:**
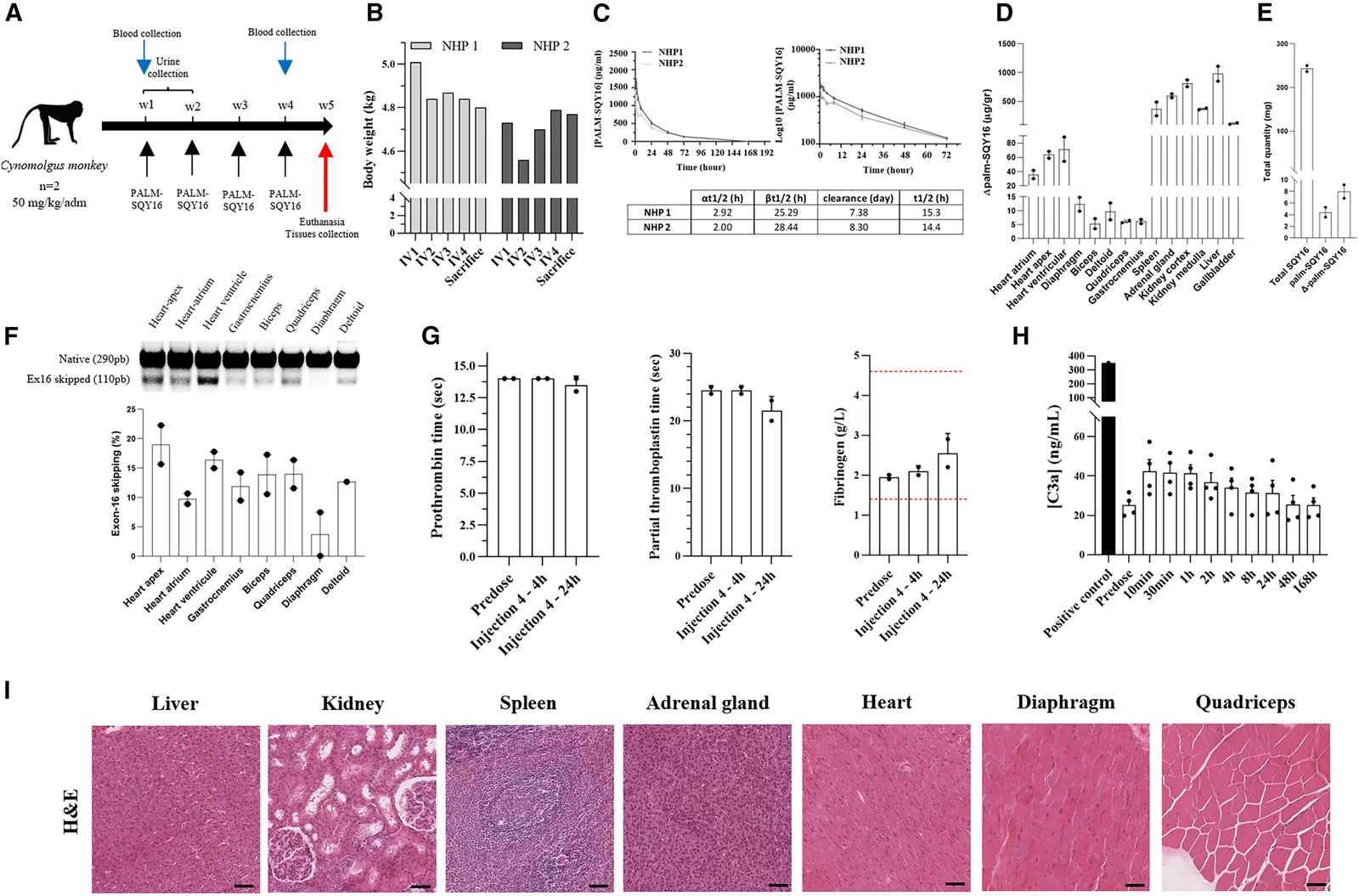
Efficacy, pharmacokinetics/dynamics, and preliminary safety evaluation of PALM-SQY16 treatment in non-human primates (A) Schematic representation of the 4-week pharmacokinetic study design in cynomolgus monkeys treated with PALM-SQY16 at 50 mg/kg/week. (B) Body weight monitoring of non-human primates (NHPs) following each intravenous (IV) injection and at study termination. (C) Serum PALM-SQY16 concentration-time profile (semi-log plot) and key pharmacokinetic parameters (α t½, β t½, clearance) following a 50 mg/kg PALM-SQY16 dose. (D) Quantification of the metabolite ΔPALM-SQY16 in various tissues after 4 weeks of PALM-SQY16 treatment (50 mg/kg/week). (E) Quantification of total SQY16 (PALM and ΔPALM), PALM-SQY16, and ΔPALM-SQY16 in urine collected over the first 24 h post-injection. (F) Detection of exon 16-skipped *dystrophin* mRNA (top) and quantification of exon 16 skipping levels (bottom) in skeletal, respiratory, and cardiac muscles following 4 weeks of PALM-SQY16 treatment (50 mg/kg/week). (G) Prothrombin time (PT), activated partial thromboplastin time (aPTT), and fibrinogen assays to evaluate the impact of PALM-SQY16 on coagulation in NHPs. (H) C3a complement activation assay in NHPs following 4 weeks of PALM-SQY16 treatment (50 mg/kg/week). (I) Hematoxylin and eosin (H&E) staining of liver, kidney, spleen, adrenal gland, skeletal, respiratory, and cardiac muscles from PALM-SQY16-treated NHPs after 4 weeks of treatment (50 mg/kg/week). Scale bars, 100 μm. Results are presented as mean ± SD; *n* = 2 NHPs.

NHPs are commonly used as translational models to evaluate drug toxicity and pharmacology prior to clinical investigations. To extend our *in vivo* findings and assess the PK/PD, efficacy, and preliminary safety profiles of PALM-SQY16 in a large animal model, we conducted a 4-week study in cynomolgus monkeys receiving weekly intravenous (IV) doses of PALM-SQY16 at 50 mg/kg (Figure 4A). This pilot investigation, conducted with a limited number of animals for ethical considerations, was designed to generate key go/no-go data before initiating more extensive and costly regulatory toxicology studies.

As illustrated in Figure 4B, PALM-SQY16 administration did not significantly affect body weight. Blood samples were collected at multiple time points on day 1 (first injection) and day 22 (fourth injection) to characterize the PK profile (predose, 10 min, 1 h, 2 h, 4 h, 8 h, 24 h, 48 h, 72 h, and 168 h; Figure 4C). Consistent with previous reports for other AONs,^35^ PALM-SQY16 plasma concentrations followed a biexponential decline, with a rapid distribution phase peaking at 10 min post-infusion, followed by a slower elimination phase. PALM-SQY16 persisted for several days in blood circulation, displaying an estimated terminal half-life of 14.9 ± 0.6 h (α t½ ≈ 2.5 ± 0.6 h; β t½ ≈ 26.9 ± 2.2 h; clearance ≈7.8 ± 0.7 days). Quantifiable levels of PALM-SQY16 remained in plasma at 27.1% ± 1.3%, 14.6% ± 0.7%, and 8.1% ± 0.6% of peak concentration at 1-, 2-, and 3-days post-infusion, respectively, with no detectable compound by day 7 (Figure 4C).

After four weeks of treatment, we quantified both PALM-SQY16 and ΔPALM-SQY16 in multiple organs (Figure 4D). While no PALM-SQY16 was detected in tissues, ΔPALM-SQY16 accumulated at high concentrations in liver (979.8 ± 185.6 μg/g, weight: 119.3 g, percent of total dose: 12.2%), kidney (cortex: 813 ± 85.8 μg/g, percent of total dose: 1.82%; medulla: 365.8 ± 17.9 μg/g, percent of total dose: 0.82%, weight: 21.5 g), adrenal gland (594.9 ± 47.9 μg/g, weight: 1.2 g, percent of total dose: 0.07%), spleen (366.2 ± 169.9 μg/g, weight: 8 g, percent of total dose: 0.31%), and gallbladder (112.1 ± 17.6 μg/g). In cardiac tissues, ΔPALM-SQY16 concentrations were 35.9 ± 8.3 μg/g in the atrium (weight: 18 g; percent of total dose: 0.07%), 63.5 ± 6.7 μg/g in the apex (weight: 18 g; percent of total dose: 0.12%), and 71.3 ± 24.2 μg/g apex (weight: 18 g; percent of total dose: 0.13%) in the ventricle, representing approximately 6.5% of hepatic levels. The metabolite was also detected in skeletal and respiratory muscles, including diaphragm (12.3 ± 3.5 μg/g), deltoid (9.8 ± 4.3 μg/g), quadriceps (6.1 ± 0.6 μg/g), gastrocnemius (6.1 ± 1.1 μg/g), and biceps (5.4 ± 2.6 μg/g), corresponding to roughly 1% of hepatic concentrations. Considering an average 7.9 ± 2.5 μg/g and a 2,000 g of total skeletal muscle in 5 kg monkey, the ΔPALM-SQY16 amount in muscle represents 1.65% of the initial total dose injected.

To assess excretion, we quantified PALM-SQY16 and ΔPALM-SQY16 in urine collected over 24 h post-injection (Figure 4E). Monkeys received an average of 243.5 ± 9.9 mg PALM-SQY16 per dose, with urinary elimination of 4.4 ± 1.2 mg PALM-SQY16 and 8.0 ± 1.6 mg ΔPALM-SQY16 corresponding to a 5.11% ± 0.03% of drug product elimination within 24 h. Together, these data define the PK profile of SQY16 (PALM and ΔPALM forms) in NHP blood and urine and confirm that the majority of the compound distributes to tissue compartments.

To demonstrate target engagement, exon 16 skipping was quantified in skeletal, respiratory, and cardiac muscles (Figure 4F). PALM-SQY16 treatment induced 11.9% ± 2.4%, 14.0% ± 2.4%, 14.0% ± 3.4%, and 12.7% exon 16 skipping in gastrocnemius, quadriceps biceps, and deltoid muscle, respectively. In cardiac tissues, skipping levels reached 19% ± 3.3%, 9.8% ± 0.9%, and 16.4% ± 1.4% in apex, atrium, and ventricle, while diaphragm exhibited 3.8% exon skipping. These results corroborate the efficacy observed in mice and confirm that PALM-SQY16 efficiently promotes exon 16 exclusion in NHP.

Safety pharmacology was evaluated in parallel, as AONs are known to potentially influence the coagulation cascade and complement pathways through protein-binding effects.^52^ We measured the extrinsic (PT) and intrinsic ( aPTT) coagulation pathways, as well as complement activation (Figures 4G and 4H). No significant changes were detected in PT, aPTT, or fibrinogen levels after four weeks of PALM-SQY16 administration, and no complement activation was observed throughout the study. Given the potential for AON accumulation in the liver and kidney, we also assessed serum biomarkers of organ injury pre- and post-treatment (Figure S4). Levels of transaminases (ALAT and ASAT), γ-GT, bilirubin, alkaline phosphatase, total protein, urea, creatinine, and glucose remained within reference ranges^53^^,^^54^ after PALM-SQY16 treatment (Figure S4A). Hematological parameters—including red blood cell count, hemoglobin, hematocrit, mean corpuscular volume, platelet count, and reticulocyte count—remain unchanged after treatment (Figure S4B). Finally, histopathological evaluation of the liver, kidney, spleen, adrenal gland, skeletal, respiratory, and cardiac muscles revealed no treatment-related lesions (Figure 4I).

Collectively, this pilot study in NHPs demonstrates that SQY16 (PALM and ΔPALM forms) displays favorable PK and biodistribution profiles, achieves meaningful exon 16 skipping in target muscles, and exhibits a reassuring preliminary safety profile. These results support further short- and long-term regulatory toxicology studies to fully assess the safety of this AON prior to clinical development.

### *Ex vivo* study in human 3D DMD muscle cultures

Preclinical development and translational research for rare DMD mutations, such as deletions in *DMD* exon 16, are limited by the absence of dedicated and physiologically relevant disease models. Recently, human three-dimensional (3D) engineered skeletal muscle systems have emerged as innovative platforms able to better recapitulate native muscle tissue architecture and function than conventional monolayer cultures.^55^

**Figure 5:**
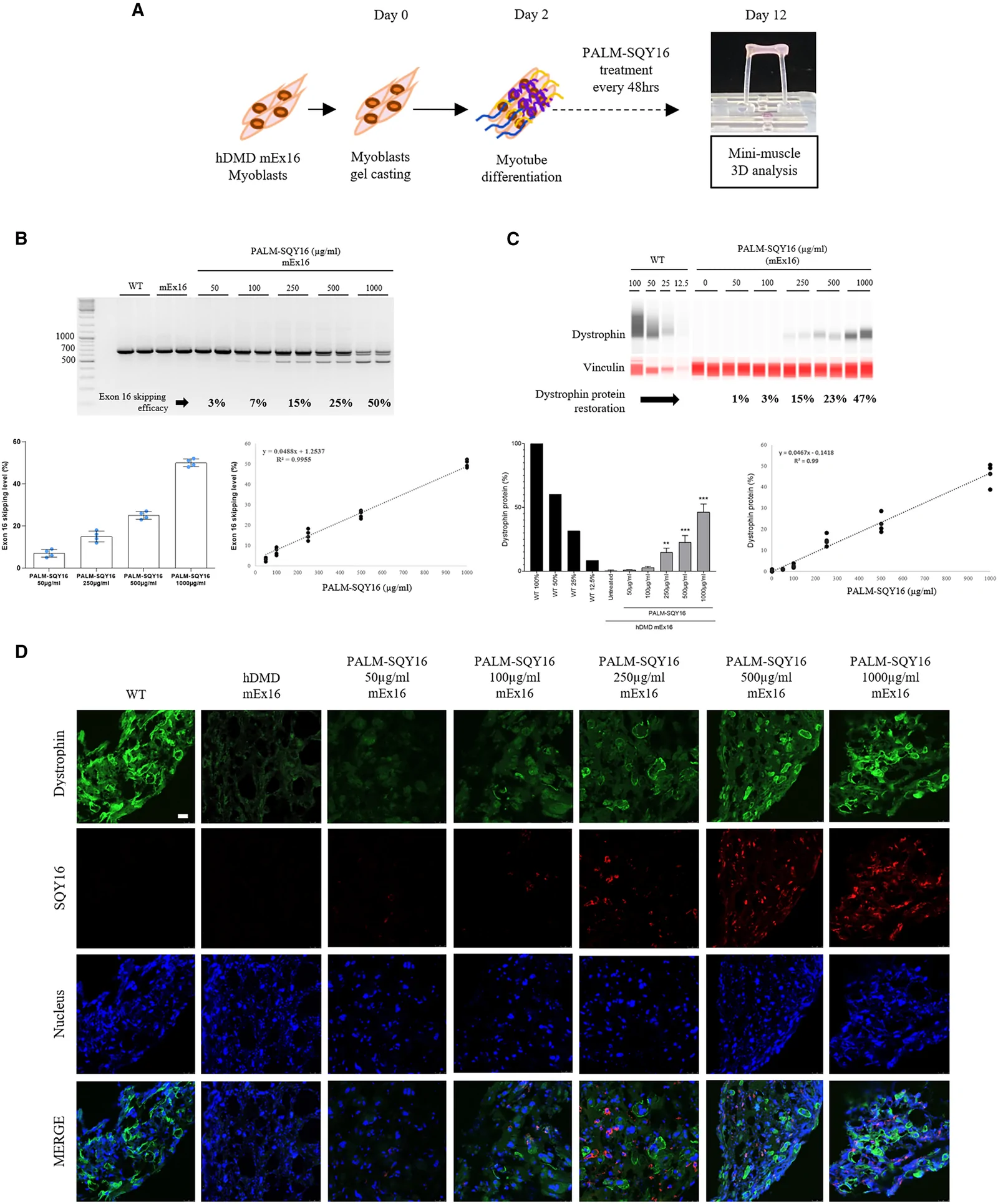
PALM-SQY16 treatment in human DMD mEx16 3D muscle cultures efficiently induces exon 16 skipping and restores dystrophin protein expression (A) Schematic overview of the generation process of 3D engineered skeletal muscle tissues. (B) Detection of exon 16-skipped *dystrophin* mRNA (top), quantification of exon 16 skipping levels (bottom left), and linear correlation between exon-skipping efficiency and PALM-SQY16 concentration (bottom right) in WT, untreated, and PALM-SQY16-treated 3D engineered muscles. (C) Dystrophin protein expression (top), vinculin-normalized dystrophin quantification (bottom left), and linear correlation between dystrophin restoration and PALM-SQY16 concentration (bottom right) in WT, untreated, and PALM-SQY16-treated 3D engineered muscles. *p* < 0.05∗, *p* < 0.01∗∗, *p* < 0.001∗∗∗; analyzed by one-way ANOVA. Results are expressed as the mean ± SD (*n* = 3 per group). (D) Co-immunostaining of WT, untreated, and PALM-SQY16-treated 3D cultures showing dystrophin (green), (PALM and ΔPALM) SQY16 probe (red), and nuclei (DAPI, blue). *n* = 3 per group. Scale bars, 50 μm.

To evaluate PALM-SQY16 efficacy under conditions closely mimicking human muscle physiology, we generated 3D engineered skeletal muscles from wild-type and hDMD mEx16 myoblasts using our established 3D culture platform designed to model muscular dystrophies and therapeutic interventions. Two days after myoblast gel casting and initiation of differentiation, hDMD mEx16 3D muscle constructs were treated every 48 h for 10 days with increasing concentrations of PALM-SQY16 (50, 100, 250, 500, and 1,000 μg/mL) without the use of any transfection reagent (Figure 5A). Tissues were harvested on day 12 for molecular and histological analyses (Figure 5A). PALM-SQY16 treatment induced a clear dose-dependent exon 16 skipping response by RT-PCR, with efficiencies of 3%, 7%, 15%, 25%, and 50% at 50, 100, 250, 500, and 1,000 μg/mL, respectively (Figure 5B). Correspondingly, dystrophin protein restoration, obtained by Jess Simple Western assay, followed a parallel dose-dependent pattern, reaching 1%, 3%, 15%, 23%, and 47% of wild-type levels at the same doses (Figure 5C). A high linearity was observed between exon skipping (R^2^ = 0.995) and dystrophin restoration (R^2^ = 0.990) across doses (250 μg/mL: 15%/15%; 500 μg/mL: 25%/23%; 1,000 μg/mL: 50%/47%) (Figures 5B and 5C). Spearman analyses between exon-skipping level and dystrophin protein restoration in human DMD 3D cultures demonstrated a strong correlation (R^2^ = 1; *p* value <0.003) (Figure S5A). To relate doses used *ex vivo* in human 3D muscle cultures to the ones used *in vivo* in animal, we quantified the amount of active metabolite form ΔPALM-SQY16 in hDMD muscle 3D cultures by QSEP100 Bio-Fragment Analyzer. After treatment of hDMD muscle 3D cultures with 250, 500, and 1,000 μg/mL doses of ΔPALM-SQY16, a respective 0.74, 4.06, and 7.72 μg/g amount of AON were quantified (Figure S6). Interestingly, the amount of ΔPALM-SQY16 (7.72 μg/g) after treatment with 1,000 μg/ml dose in 3D mini-muscle falls in the range of the 50 mg/kg regimen used in treated NHP skeletal muscles (7.94 μg/ml). Consistent with molecular analyses, immunofluorescence staining of 3D muscle sections confirmed a dose-dependent SQY16 signal (PALM and ΔPALM) and subsequent restoration of dystrophin expression localized at the sarcolemma of treated myofibers (Figure 5D).

In summary, these results demonstrate that PALM-SQY16 efficiently induces exon 16 skipping and restores dystrophin protein expression in a robust, dose-dependent manner in human 3D engineered DMD skeletal muscle, further supporting its translational therapeutic potential.

### Molecular benefits of dystrophin restoration following PALM-SQY16 treatment

**Figure 6:**
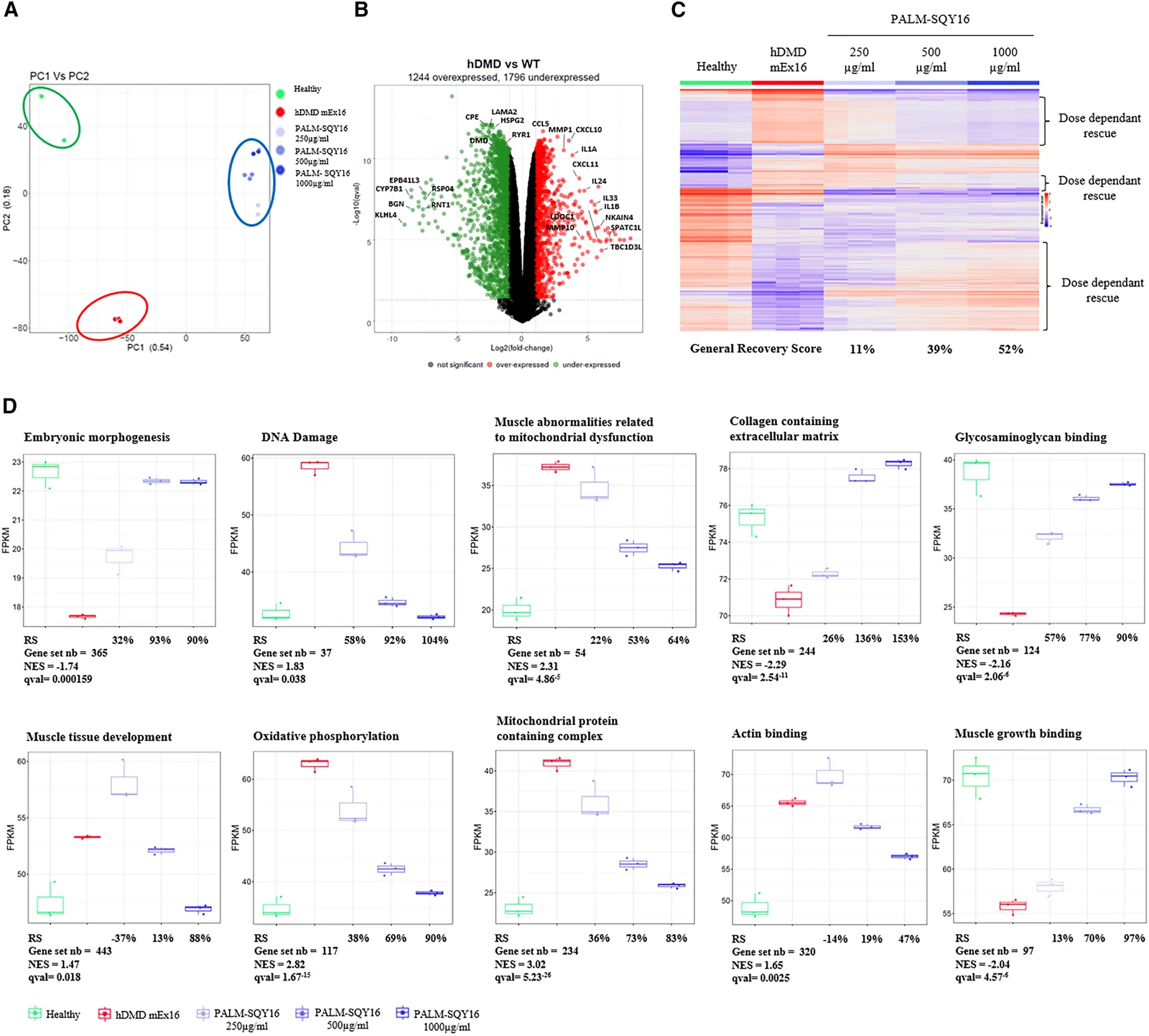
Transcriptomic profiling of wild-type, untreated, and PALM-SQY16-treated hDMD mEx16 3D muscle cultures (A) Principal component analysis (PCA) illustrating the transcriptomic clustering of healthy, untreated, and PALM-SQY16-treated 3D muscle cultures. (B) Volcano plot highlighting significantly dysregulated genes in hDMD mEx16 3D cultures compared to healthy. Green and red dots represent significantly downregulated (≤–2-fold, *q* value <0.05) and upregulated (≥2-fold, *q* value <0.05) genes, respectively. (C) Heatmap depicting expression profiles across healthy, untreated, and PALM-SQY16-treated 3D cultures for 3,040 previously defined DMD-dysregulated genes. Red indicates upregulation, and blue indicates downregulation. The Global Recovery Score reflects the degree of transcriptomic normalization across all DMD-dysregulated genes. (D) Overview of key dysregulated pathways and the beneficial transcriptional modulation observed following PALM-SQY16 treatment. Results are expressed as the mean ± SD (*n* = 3 per group).

To investigate the molecular consequences of dystrophin restoration induced by PALM-SQY16, we performed RNA sequencing (Illumina platform) on healthy, untreated hDMD mEx16, and PALM-SQY16-treated (250, 500, and 1,000 μg/mL) 3D human muscle cultures. We first analyzed transcriptomic dysregulation in untreated hDMD mEx16 3D cultures compared with healthy controls (Figures 6A and 6B). Principal component analysis (PCA) revealed clear separation between healthy and hDMD samples, indicating distinct transcriptional profiles and strong intra-group homogeneity (Figure 6A). Among the 3,040 genes differentially expressed in hDMD compared with healthy cultures, 1,244 were upregulated and 1,796 were downregulated (FPKM ≥1; |fold change| ≥2; q ≤ 0.05) (Figures 6B and 6C). As expected, *DMD* transcript levels were significantly reduced in hDMD samples relative to healthy (Figure 6B). Notably, several inflammation-related genes were markedly overexpressed, including members of the interleukin family (IL1A, IL1B, IL24, and IL33), interferon-γ-induced chemokines (CXCL10 and CXCL11), and the pro-inflammatory CCL5. Additional upregulated genes included SSP1 (a component of the dystrophic inflammatory environment), CTSL (a myofibril necrosis marker), and LDOC1, a regulator of the NF-κB pathway—a key mediator of inflammation in dystrophic muscle^56^ (Figure 6B; Table S3). Genes associated with cytoskeletal dynamics and ion transport were also dysregulated, including SPATC1L (actin polymerization/depolymerization), NKAIN4 (Na^+^/K^+^-ATPase modulation), and TBC1D3L (GTPase activator signaling). Altered expression of key dystrophic markers was observed in metabolic pathways (CYP7B1 and CPE), cytoskeletal integrity (RSPO4), endoplasmic reticulum and membrane trafficking (RTN1), and excitation-contraction coupling (RyR1) (Figure 6B; Table S3). Consistent with known DMD pathology,^57^ we also observed profound extracellular matrix (ECM) remodeling, with significant reductions in transcripts encoding laminin subunit alpha 2 (LAMA2), fibronectin 1 (FN1), biglycan (BGN), perlecan (HSPG2), and the actin-associated protein KLHL4. Collectively, these data confirm that hDMD mEx16 3D cultures recapitulate multiple molecular hallmarks of dystrophic muscle.

We next assessed whether PALM-SQY16 treatment could rescue this dysregulated transcriptomic signature toward a healthy profile. The expression of the 3,040 dysregulated genes was visualized on a heatmap comparing untreated hDMD, PALM-SQY16-treated (250, 500, and 1,000 μg/mL), and healthy 3D muscle cultures (Figure 6C). To quantitatively evaluate transcriptomic rescue, we applied a recovery score (RS) metric following the calculation RS= (“treated” − “untreated”)/(“normal” − “untreated”) ∗100] = [(PALM-SQY16 treated hDMD 3D − untreated hDMD 3D)/(Healthy 3D – untreated hDMD 3D) ∗ 100].^58^ The RS ranges from 0% representing the untreated dystrophic condition (absence of functional dystrophin) and 100% corresponds to the healthy state. PALM-SQY16 treatment progressively shifted the transcriptome toward the healthy profile, with recovery scores of 11%, 41%, and 52% following 250, 500, and 1,000 μg/mL treatments, respectively (Figure 6C). Interestingly, multi-matrix Pearson analysis demonstrated a high correlation between exon-skipping level, dystrophin and transcriptomic rescues in 3D muscle cultures following PALM-SQY16 treatment (Figure S5B). At the pathway level, Gene Ontology (GO) analysis confirmed dose-dependent normalization of multiple biological processes, including muscle development and morphogenesis (e.g., muscle tissue development, embryonic morphogenesis, and actin binding), mitochondrial biogenesis and function (mitochondrial protein-containing complex and oxidative phosphorylation), and extracellular matrix remodeling (collagen-containing ECM and glycosaminoglycan binding). Additional improvements were observed in pathways related to DNA damage repair and cellular stress response (Figure 6D).

Overall, this set of data demonstrates that PALM-SQY16 treatment restores the transcriptomic landscape of hDMD mEx16 3D engineered muscle toward the healthy profile in a dose-dependent manner. Importantly, it should be noted that if this transcriptomic analysis, aiming to assess PALM-SQY16 efficacy on dysregulated dystrophic transcripts, precludes to investigate specific changes induced by the AON, complementary *in silico* off-target profiling will be necessary to reinforce this set of data.

### Exon skipping-mediated dystrophin restoration ameliorates the dystrophic proteomic profile

Numerous studies have revealed that transcriptomic and proteomic data frequently exhibit weak correlation, owing to complex post-transcriptional, translational, and degradation processes.^59^ To complement our transcriptomic analyses and assess the impact of dystrophin restoration at the protein level, we performed quantitative mass spectrometry-based proteomic profiling of healthy, untreated hDMD mEx16, and PALM-SQY16-treated (250, 500, and 1,000 μg/mL) 3D human muscle cultures.

**Figure 7:**
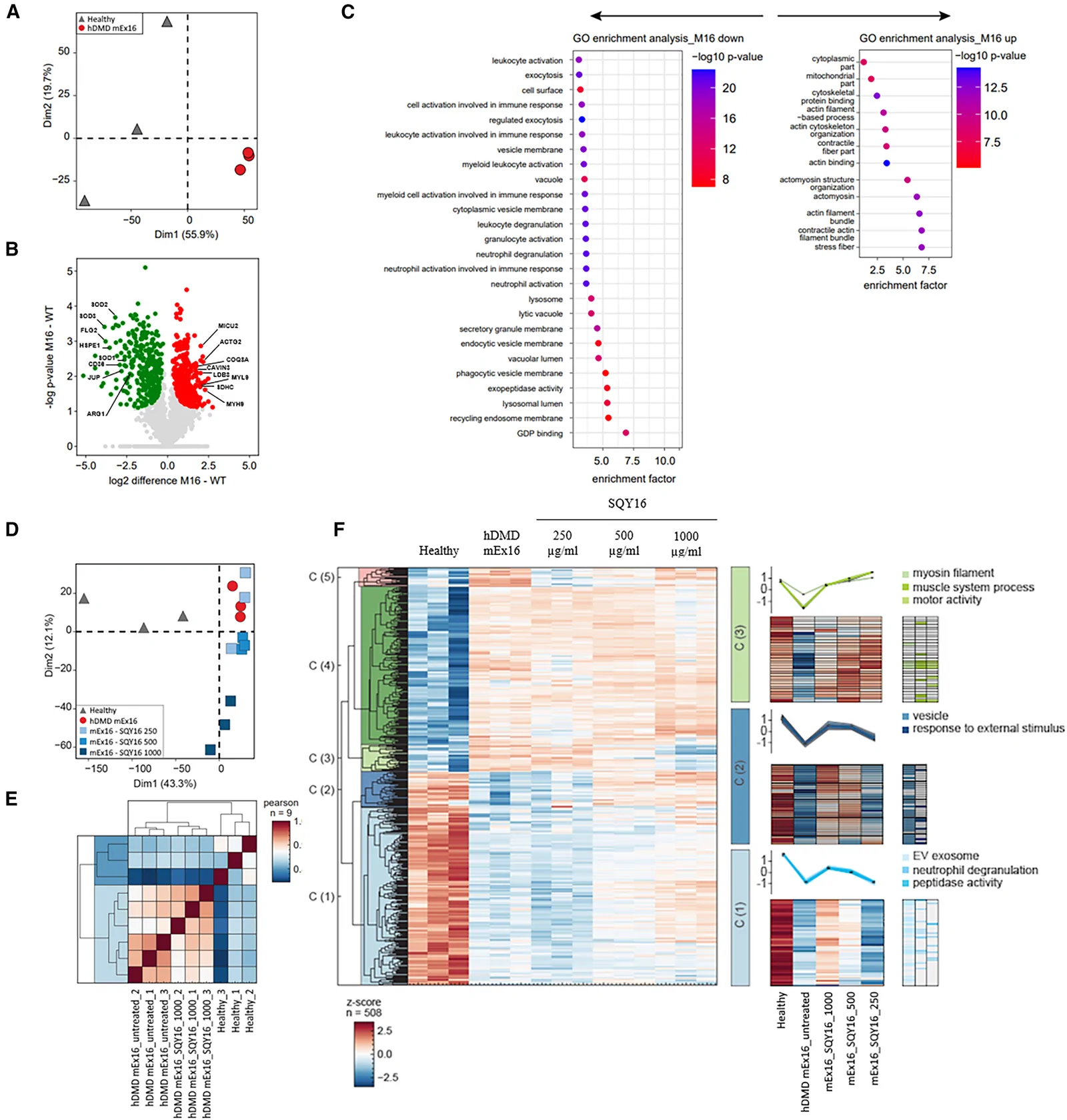
PALM-SQY16 treatment partially restores the dysregulated proteomic landscape in hDMD mEx16 3D engineered skeletal muscles (A) Principal component analysis (PCA) of the proteomes from healthy and untreated 3D cultures. (B) Volcano plot showing significantly dysregulated proteins in hDMD mEx16 3D cultures compared to healthy. Green and red dots indicate significantly downregulated (≤−1.25-fold, *q* value <0.05) and upregulated (≥1.25-fold, *q* value <0.05) proteins, respectively. (C) Protein ontology analysis highlighting the most significantly downregulated (left) and upregulated (right) protein pathways. (D) PCA of proteomes from healthy, untreated, and PALM-SQY16-treated 3D cultures. (E) Clustering of proteomic profiles from healthy, untreated, and PALM-SQY16 (1,000 μg/mL)-treated hDMD mEx16 3D cultures. (F) Hierarchical clustering of significantly altered proteins across all experimental groups. Red indicates upregulated and blue indicates downregulated proteins. The Global Recovery Score represents the overall normalization of the 682 DMD-dysregulated proteins following PALM-SQY16 treatment. Key protein pathways showing the strongest recovery are indicated for each group. Results are expressed as the mean ± SD (*n* = 3 per group).

Consistent with the transcriptomic results, PCA revealed a clear separation between healthy and hDMD samples, underscoring distinct proteomic signatures (Figure 7A). Among the 5,539 proteins detected, 682 were significantly dysregulated in hDMD compared with healthy cultures, including 345 upregulated (log 2 difference ≥0.5; q ≤ 0.05) and 337 downregulated (log 2 difference ≤ −0.5; q ≤ 0.05) proteins (Figure 7B). GO enrichment analyses (Figure 7C; Table S4) identified major alterations in proteins associated with stress fiber organization (LDB3, PDLIM3, MYH9, and MYL9) and actin cytoskeleton regulation (DPYSL3, PASCIN1, CAVIN3, and PDLIM3). Several proteins involved in negative regulation of actin filament severing (CAVIN3 and MYH9) and muscle contractile function (ACTN, PDLIM3, MYH9, and MYL9) were profoundly dysregulated. Additional perturbations were observed in proteins related to mitochondrial homeostasis (MICU2, C5O, and COQ8A) and endoplasmic reticulum biogenesis (SDCH). Interestingly, proteins involved in secretory vesicle dynamics (SOD1, ARG1, CD36, JUP, FLG2, and S100A7) and extracellular exosome function (SOD1, SOD2, SOD3, GLRX, and HSPE1) were markedly downregulated, indicating broad defects in exocytosis, endocytosis, and membrane trafficking within hDMD mEx16 3D muscles. Overall, these results confirm substantial proteomic remodeling in dystrophic engineered muscle, in part aligned with transcriptomic alterations.

We next evaluated the restorative effects of PALM-SQY16 treatment across the dysregulated DMD proteome. PCA revealed a dose-dependent shift of PALM-SQY16-treated hDMD samples toward the healthy cluster, with increasing separation from untreated dystrophic samples at higher PALM-SQY16 concentrations (Figure 7D). Hierarchical clustering placed PALM-SQY16-treated hDMD proteomes intermediate between untreated hDMD and healthy profiles (Figure 7E). Among the 682 dysregulated proteins identified in hDMD cultures, PALM-SQY16 treatment produced progressive recovery with general recovery scores of 5%, 17%, and 31% at 250, 500, and 1,000 μg/mL, respectively. Of interest, it should be noted that a high correlation between exon-skipping level, dystrophin and transcriptomic as well as proteomic rescues in 3D muscle cultures following PALM-SQY16 treatment was observed by multi-matrix Pearson analysis (Figure S5B). GO enrichment and cluster analyses demonstrated that PALM-SQY16 partially normalized several key functional modules in a dose-dependent manner, notably proteins involved in endosome organization (cluster C1), vesicle biogenesis (cluster C2), and muscle system processes and motor activity (cluster C3) (Figure 7F).

Taken together, these findings indicate that PALM-SQY16 treatment progressively ameliorates the dystrophic proteomic signature of hDMD mEx16 3D skeletal muscle in a dose-dependent fashion, mirroring transcriptomic recovery. These results underscore benefits of dystrophin restoration following PALM-SQY16-mediated exon 16 skipping, extending beyond RNA correction to the proteomic level.

## Discussion

To date, half of the eight FDA-approved drugs for DMD are exon-skipping AON therapies.^14^ While 90.3% of DMD patients carry mutations or deletions that could theoretically be targeted by one of 115 exon-skipping strategies,^19^ the four currently approved AONs apply to only ∼27% of patients. Consequently, the majority of individuals with DMD remain without an exon-skipping therapeutic option. Despite these initial successes, the substantial time, cost, and technical effort required for the design and preclinical validation of personalized AONs continue to limit access for eligible patients.^60^

To accelerate drug discovery and streamline the transition from design to clinical translation, we propose here a faster and more efficient methodology for identifying AONs suitable for clinical use. The approach focuses on prioritizing tasks from the start and rather than selecting only the most effective AON, to identify compounds with the fewest clinical risks. After high-throughput screening, selected AONs are tested *in vitro* for compatibility with intravenous delivery and for their interactions with blood proteins across different species to determine the most relevant animal models for predicting human pharmacokinetics. Elimination of unsuitable AONs before animal testing is critical to reduce both research time and costs and avoid unnecessary animal studies.

To illustrate the approach, we focused on the identification of a PALM-tcDNA compound targeting a rare *DMD* exon 16 mutation. We designed and synthesized 59 PALM-tcDNAs spanning exon 16. Although *in silico* design guidelines^61^ and predictive modeling approaches^62^ could potentially reduce the number of candidates, all 59 AONs were subjected to high-throughput screening using an ICW assay. This 96-well immunofluorescent method quantifies dystrophin restoration in myotube cultures and allows for large-scale, quantitative assessment of AON activity.^48^ While many studies primarily assess AON efficacy by measuring exon-skipped transcripts, we selected dystrophin protein restoration as the primary screening endpoint. Restoration of dystrophin necessarily implies successful exon skipping, whereas high transcript correction does not always translate into functional protein rescue. Indeed, discrepancies between mRNA and protein outcomes have been documented in both preclinical and clinical settings. For example, in animal studies, locked nucleic acid (LNA) AONs induced robust exon 53 skipping (up to 90%) without detectable dystrophin protein restoration,^63^ and in patients, the FDA-approved exon 53-skipping drug viltolarsen increased exon skipping by 43.9% at the mRNA level but achieved only 5.9% of normal dystrophin expression.^64^ Such inconsistencies likely stem from differences in *DMD* transcript balance,^65^ stability of truncated pseudo-dystrophins, post-transcriptional regulation, or assay-dependent transcript amplification artifacts. Therefore, our screening methodology prioritizes protein-level screening as the primary criterion, followed by transcript-level confirmation and *in vitro* safety counter-screens to eliminate candidates with undesirable effects. Of the initial 59 PALM-tcDNAs, two candidates efficiently restored dystrophin protein; both confirmed exon 16 skipping at the RNA level. Only one—AON-5—namely PALM-SQY16, met all safety criteria, exhibiting an optimal *in vitro* pharmacological profile.

With more than 60 dystrophin-deficient animal models available,^66^ DMD research benefits from a wide array of preclinical systems, each with distinct strengths and limitations. Mouse models, such as *mdx*,^67^ hDMD,^50^ and *mdx*-hDMDΔ52^68^ have been instrumental in developing several authorized treatments. However, the absence of animal models for rare mutations—such as exon 16 deletions—impedes preclinical testing and clinical translation. Since no existing DMD model carries an exon 16 defect, we conducted exploratory PK, PD, and safety studies in wild-type mice and healthy NHPs. Following PALM-SQY16 treatment, mice and NHPs exhibited comparable biodistribution profiles, with the highest accumulation in the kidney and liver, followed by heart, diaphragm, and other skeletal muscles—consistent with previous tcDNA data.^35^ In mice, PALM-SQY16 displayed a 3.23-h terminal half-life and a 1.25-day clearance, while in NHPs, these values extended to 14.9 h and 7.8 days, respectively. These species differences correlate with the degree of PALM-SQY16 binding to serum albumin, as albumin interaction enhances tcDNA stability and potency.^35^ Importantly, PALM-SQY16 binding affinity was similar in monkey and human sera, suggesting translatable PK/PD behavior between the two species. In mice, PALM-SQY16 induced 14%–25% exon 16 skipping across skeletal, respiratory, and cardiac muscles—comparable to effects observed in NHPs. The monkey pilot study, despite limitation in sample size for ethical reasons, provided essential preliminary safety data supporting the absence of overt toxicity. Together, these results justify progression to regulatory toxicology studies, a necessary step toward clinical trials.

Beyond animal models, 3D human skeletal muscle cultures have recently emerged as physiologically relevant platforms for modeling muscle disorders and therapeutic interventions.^69^ Derived from patient myoblasts, these microtissues recapitulate the architecture, contractile maturation, and disease hallmarks of DMD.^55^ Combined with CRISPR-Cas9-based genome editing, such systems enable the generation of isogenic dystrophic lines, offering unprecedented flexibility for evaluating genotype-specific therapies.^70^^,^^71^ In this study, we developed 3D muscle cultures from hDMD mEx16 myoblasts to compensate for the lack of suitable disease and animal models. PALM-SQY16 treatment induced dose-dependent exon 16 skipping, dystrophin restoration, and normalization of transcriptomic and proteomic profiles toward wild-type levels. Strong correlations were observed between PALM-SQY16 dose, exon-skipping efficacy, dystrophin recovery, and molecular rescue. While 3D skeletal muscle technology for DMD remains in its infancy, it represents a transformative step toward personalized preclinical testing. Future refinements—such as incorporation of multiple cell lineages and vascular or immune components—will further enhance physiological relevance.^72^ Nonetheless, simplified single-lineage systems, as used here, provide valuable mechanistic insights unconfounded by systemic effects such as contraction, perfusion, or immune modulation.

The overarching goal of preclinical and non-clinical studies is to establish a robust therapeutic window, predict pharmacokinetic and pharmacodynamic in patients, and anticipate the drug risk-benefit profile to ensure both efficacy and safety in humans. Consequently, it is essential to relate doses used in preclinical studies and consider future dosing in patients. Our data revealed that PALM-SQY16 exhibits comparable binding affinity in monkey and human sera, suggesting translatable PK/PD properties between the two species. Furthermore, treatment of human DMD 3D mini-muscles with 1,000 μg/mL PALM-SQY16 every two days with medium change for 14 days resulted in similar levels of the active metabolite, ΔPALM-SQY16, than in skeletal muscle tissue after a four week treatment with a weekly 50 mg/kg PALM-SQY16 administration in NHPs. Although caution is warranted when extrapolating across experimental contexts, these findings suggest that the 1,000 μg/mL regimen used in *ex vivo* studies may relate to the 50 mg/kg regimen in NHPs and consequently that the 500 and 250 μg/mL regimens may correspond to the 25 and 12.5 mg/kg doses, respectively. In human DMD 3D mini-muscles, treatment with the 500 μg/mL resulted in a 25% exon 16 skipping efficiency and 23% dystrophin protein restoration translated in a respective 39% and 18% recovery of dystrophic transcriptomic and proteomic profiles toward healthy levels. In an appropriate human dystrophic context, the 500 μg/mL-25 mg/kg regimen may therefore be considered therapeutically relevant, given that 15%–20% dystrophin restoration is considered as sufficient to largely prevent disease progression.^73^^,^^74^ In the ongoing AVANCE1 phase 2 clinical trial in DMD patients, PALM-SQY51 is administered intravenously at doses of 10, 16, or 25 mg/kg once weekly for four weeks, followed by a one-month break, repeated four times. Whereas novel administration routes as under investigation for tcDNA delivery^75^ and that future tcDNA clinical development will undoubtedly benefit from insight and the lessons learned in the first-in-human AVANCE1 clinical trial with the PALM-SQY51, we can envision that future clinical studies— including *n* = 1 trials targeting very small patient populations such as exon 16-skipping—may adopt similar study designs. Nevertheless, despite these extrapolations and considerations, it is important to emphasize that clinical dose selection ultimately relies on mandatory regulatory toxicology studies defining the no observed adverse effect level (NOAEL).

Historically, dystrophin has been regarded primarily as a mechanical stabilizer, anchoring the cytoskeleton to the extracellular matrix and protecting the sarcolemma during contraction.^7^ However, emerging evidence suggests broader non-mechanical functions, including roles in signal transduction,^76^^,^^77^ vascular regulation,^78^ neural function,^79^ in brain^80^ and stem cell homeostasis.^9^^,^^81^ In our 3D model, PALM-SQY16-mediated dystrophin restoration led to parallel, dose-dependent recovery of transcriptomic and proteomic networks. At low PALM-SQY16 doses (15% exon skipping, 15% dystrophin rescue), transcriptomic and proteomic recovery reached 11% and 5%, respectively, whereas the highest dose (50% exon skipping, 47% dystrophin restoration) yielded 52% and 31% molecular recovery. Gene and protein clusters involved in embryonic morphogenesis, muscle development, mitochondrial biogenesis, and vesicle trafficking were markedly rescued, alongside canonical pathways governing muscle contraction and cytoskeletal organization. These findings, consistent with prior reports,^8^^,^^81^^,^^82^ support the concept of dystrophin as a morphogenetic conductor acting beyond its mechanical role. Dystrophin deficiency has been associated with dysregulation of junctional proteins critical for embryonic somitogenesis^83^ and with transcriptional perturbations in muscle development genes,^82^ reinforcing the view of DMD as both a developmental and metabolic disorder that manifests before muscle maturation. While our results strengthen this hypothesis, further studies are warranted to clarify the morphogenetic functions of dystrophin during myogenesis.

In summary, we presented a methodology for the rapid identification of relevant AONs through early candidate prequalification and the elimination of unsuitable leads prior to animal testing, thereby reducing both development time and costs. Selected AONs were assessed *in vitro* at an early stage for compatibility with intravenous delivery, and their species-specific serum protein-binding profiles were characterized to enable the selection of the most relevant animal and disease models for predicting human pharmacokinetics. This proof-of-concept study, exemplified by the discovery and characterization of PALM-SQY16, demonstrated the feasibility of developing an effective and safe lead compound within a reasonable time frame and at manageable cost, with the view to make possible future *n* = 1 trials for very small DMD populations.

## Materials and methods

### ASOs and animal experiments

All animal procedures were performed in compliance with national and European regulations and approved by the French Ministry of Higher Education and Research (authorization no. APAFIS #34354-2021121417092550 v4). Transgenic human DMD mice were imported from the Leiden University Medical Center,^50^ bred in our animal facility (Plateforme 2Care, UFR des Sciences de la Santé, Université de Versailles Saint-Quentin) and maintained under standard housing conditions (12 h light/dark cycle) with *ad libitum* access to food and water.

Adult hDMD mice (mdx, C57BL/10ScSc-Dmdmdx/J, males, 6–8 weeks old) received intravenous injections of AONs in the retro-orbital sinus once weekly, as specified in the results section, under isoflurane anesthesia. Experiments on cynomolgus monkeys were conducted at the “Commissariat à l’Énergie Atomique et aux Énergies Alternatives” (CEA, Fontenay-aux-Roses, France) in accordance with approved institutional and governmental guidelines. Adult healthy monkeys were injected intravenously via a saphenous vein catheter connected to an ambulatory infusion pump, once weekly under zoletil anesthesia. Blood samples were collected at multiple time points, and urine was collected over 24 h using metabolic cages. Urine samples were stored at −80°C until analysis. Animals were euthanized one week after the final AON injection, and tissues were harvested, snap-frozen in liquid nitrogen-cooled isopentane, and stored at −80°C.

Sample sizes and *n* values are provided in each figure legend. Experiments were not randomized.

All tcDNA-AONs targeting exon 16 of the human *dystrophin* pre-mRNA (detailed in Figure S3) were synthesized by SQY Therapeutics. Palmitic acid was conjugated to the 5′ end of tcDNA-PO AON via a C6-amino linker and a phosphorothioate moiety.

### Cell culture

Human wild-type (wt) AB1190 cells are immortalized myoblasts derived from a healthy donor biopsy, while DMD-Hex16 cells are immortalized myoblasts carrying a *DMD* mutation (codon #659 of exon 16: del_AA). Both lines were obtained from the MyoLine Immortalization Platform (Myology Institute, Paris).^84^ Cells were cultured in Skeletal Muscle Cell Growth Medium (PromoCell, C-23060) supplemented with 12.5% bovine serum albumin (Eurobio, CVFSVF00-01) at 37°C with 5% CO_2_. Cells were seeded in 6-well plates (4.2 × 10^5^ cells/well) or 96-well plates (1.4 × 10^4^ cells/well) for RT-PCR/Simple Western and ICW analyses, respectively. After 24 h, differentiation was induced with Skeletal Muscle Cell Differentiation Medium (PromoCell, C-23061). TcDNA oligonucleotides were transfected using the Lipofectamine LTX & Plus Reagent kit (Invitrogen, 15338-100) according to the manufacturer’s protocol. After four days of differentiation and transfection, myotubes were collected for analysis.

3D artificial muscles were generated as previously described^72^ using 10% Matrigel (BD 356230) and 3.5 mg/mL human fibrinogen (Tissucol Duo 500, Baxter; Sigma-Aldrich F8630), polymerized with 3 U/mL thrombin (Biopur 10-13-1104; Sigma-Aldrich T7326). A total of 1 × 10^6^ immortalized myoblasts were mixed with the matrix cocktail (120 μL total volume) and polymerized between two silicone posts (EHT Technologies, Hamburg).

After polymerization, gels were maintained in growth medium (PromoCell, C-23060) supplemented with 33 μg/μL aprotinin (Sigma, A3428) to prevent fibrin degradation. After 48 h, gels were transferred to differentiation medium (PromoCell, C-23061) with aprotinin and maintained up to day 14, with medium changes every 2 days.

### Dystrophin quantification

For the Jess Simple Western assay, proteins were extracted from 3D artificial muscles using RIPA buffer (Thermo Fisher Scientific, 89900) supplemented with protease inhibitors (Thermo Fisher Scientific, A32963). Protein concentration was determined with the BCA Protein Assay Kit (Thermo Fisher Scientific, 23227). Samples were prepared using the 66–440 kDa Separation Module (ProteinSimple, Bio-Techne SM-FL005). Briefly, proteins (1 mg/mL) were diluted in 1× fluorescent master mix (ProteinSimple PS-ST01EZ-8) and denatured at 95°C for 5 min. Primary antibodies included mouse monoclonal anti-dystrophin (DYS1; NCL-Leica, 1:50) and rabbit polyclonal anti-vinculin (Abcam, ab73412, 1:1000). Detection was performed using the Anti-Mouse and Anti-Rabbit HRP modules (ProteinSimple, Bio-Techne DM-002 and DM-001 respectively). For ICW assays (LI-COR, 926–19156), cells were fixed with ice-cold methanol for 10 min, permeabilized with 0.1% PBS-Tween for 5 min, and blocked for 2 h at room temperature in Intercept (TBS) Blocking Buffer (LI-COR, 927–60001). Primary antibodies (mouse DYS1; NCL-Leica,1:50; rabbit anti-α-actinin; Thermo Fisher Scientific #701914, 1:500) were incubated overnight at 4°C, followed by fluorescent secondary antibodies IRDye 800CW anti-mouse and IRDye 680RD anti-rabbit (LI-COR, 926–32350 and 926–68073, respectively, 1:800).

### RNA analysis

Total RNA was extracted from cells or cryosectioned muscle using TRIzol reagent (Thermo Fisher Scientific, 15596026). Reverse transcription was performed from 500 ng total RNA using the SSIII Reverse Transcriptase System (Thermo Fisher Scientific, 18080044). PCR was carried out with Q5 HF polymerase (New England Biolabs, M0491) using the following primers: Ex15F (5′-CAAGTCTTCAAAAACTGGCC-3′) and Ex17R (5′-GATTTGCTCCCTTGTGGTC-3′) for monkey study and Ex14F (5′-GTATTGGGAGATCGATGGG-3′) and Ex18R (5′-AGTCTGAGAAGTTGCCTTCC-3′) for *in vitro* and mouse studies. PCR products were visualized on a 2% agarose gel, extracted and purified and then sent for Sanger sequencing (Genewiz, Leipzig, Germany).

### *In vitro* toxicity assessments: Dimerization, flocculation, coagulation and complement C3a activation

Human serum and plasma from healthy volunteers were obtained from the French Blood Donor Organization (EFS, *Établissement Français du Sang*). For the dimerization assay, 4 μg of tcDNA were loaded with glycerol onto a NuPAGE Tris-acetate 7% gel (Thermo Fisher Scientific, EA0358BOX) according to the manufacturer’s instructions. Electrophoresis was carried out for 1 h 30 at 90 V, followed by staining with diluted Stain-All solution (Sigma, E9379). Gels were scanned using an Epson scanner (Epson, Perfection V800 Photo, B11B223401). Positive control is a full phosphorothioate tc-DNA (5'-AAGATGGCATTTCTA-3') exhibiting sequence-dependent toxicity due to homodimer formation. Flocculation assay was performed using tcDNA samples mixed with human or monkey EDTA plasma to a final concentration of 2 mg/mL. Optical density at 595 nm was monitored for 60 min using a FluoStar Omega Microplate Reader (BMG Labtech). Positive control is a full phosphorothioate tc-DNA (5′-AAGATGGCATTTCTA-3') exhibiting sequence-dependent toxicity due to homodimer formation. For coagulation assays, human or monkey citrated plasma was incubated *in vitro* with 0.2 or 1 mg/mL tcDNA for 30 min at 37°C. PT and aPTT were then determined using a semi-automated START Max coagulometer (Stago) according to the manufacturer’s recommendations. To study complement C3a activation, tcDNA was incubated with human or monkey serum for 45 min at 37°C. Positive control is a full phosphorothioate tc-DNA (5′-AAGATGGCATTTCTA-3′) activates coagulation and compliment C3a. Complement activation was quantified using the Human C3aPlus kit (Quidel Corp., San Diego, CA, USA, A031) according to the supplier’s instructions.

### Serum analyses

Serum parameters, including alanine aminotransferase (ALAT), aspartate aminotransferase (ASAT), γ-glutamyl transferase (γGT), alkaline phosphatase (ALP), total bilirubin, creatinine, urea, total protein, glucose, red blood cell count, hemoglobin, hematocrit, mean corpuscular volume, hemoglobin concentration, white blood cell count, platelet number, and reticulocyte levels, were analyzed by Anydiag laboratory (Gentilly, France). For serum protein capture and proteomic analysis, biotinylated tcDNA (3′ end) was incubated with 20 μL of high-capacity streptavidin agarose resin beads (Thermo Fisher Scientific, 20361). After PBS washing, human, monkey, or mouse serum was added to the tcDNA-bead complex and incubated for 30 min at room temperature. Following additional PBS washes and centrifugation, half of the bead suspension was sent for proteomic analysis (ProtéoSeine, Institut Jacques Monod, Paris, France). The remaining beads were denatured at 100°C for 5 min and separated on NuPAGE Bis-Tris 4%–12% gels (Thermo Fisher, NP0321BOX). Gels were stained with ReadyBlue Protein Gel Stain (Sigma, RSB-1L) for 1 h in the dark and scanned using an Epson imaging system (Epson, Perfection V800 Photo, B11B223401).

### tcDNA quantification in sera, urines, and tissues

Tissues were homogenized using a Precellys 24 homogenizer (Bertin Instruments, France) in lysis buffer (100 mmol/L Tris-base pH 8.5, 200 mmol/L NaCl, 5 mmol/L EDTA, 0.2% SDS) containing 2 mg/mL proteinase K (Thermo Fisher Scientific, 25530049) at a ratio of 50 mg tissue per mL of buffer. Samples were incubated overnight at 55°C, centrifuged at 14,000 rpm for 15 min (Sorval ST 8R, rotor 75005719), and supernatants were used for analysis. For serum and urine, 25 μL of sample were incubated overnight at 55°C with 75 μL of the same lysis buffer containing 2 mg/mL proteinase K. tcDNA-AONs were extracted by solid-phase extraction (SPE) using Oasis HLB 1cc (10 mg) cartridges (Waters, 186000383). Serum quantification was performed by LC-MS on an Agilent 1100 HPLC system coupled to an Agilent 6530 TOF-MS. Standard curves for PALM-SQY16 and ΔPALM-SQY16 were generated using six concentration points. Separation was achieved on an ACQUITY UPLC Oligonucleotide BEH C18 column (130 Å, 1.7 μm, 2.1 × 100 mm, Waters, 186003950) maintained at 75°C. The mobile phases consisted of 400 mM HFIP and 15 mM TEA with 10% methanol (A) and methanol (B), at 0.2 mL/min. Two gradient programs were applied to separate unconjugated and palmitoylated AONs. Peaks were identified by comparison with theoretical m/z tables, and quantification was based on UV chromatogram peak areas calibrated against standard curves. Urine and tissue quantification were performed using the QSEP100 Bio-Fragment Analyzer (Bioptic). Standard curves for PALM-SQY16 and ΔPALM-SQY16 were established over six concentration points. Samples were mixed with dilution buffer and a fluorescent probe complementary to the tcDNA sequence (5′-Alexa488–ATTCTGTGATCTTTCTTGTT-3′, Eurogentec). Separation was carried out with 4 kV injection (20 s) and 6 kV migration (180 s). Peak areas were measured, and tcDNA concentrations were calculated using calibration curves.

### Immunohistochemistry analysis

Hematoxylin and eosin (H&E) staining was performed on 8 μm transverse cryosections from paraffin-embedded tissues stored at +4°C. Staining followed standard protocols.^85^ Whole muscle sections were scanned using an Axioscan Z1 automated slide scanner (Zeiss, Germany) equipped with ZEN 2.6 software and a Plan APO 10×/0.45 NA objective.

### Immunostaining of whole 3D artificial muscles

Prior to cryo-embedding, 3D artificial muscles were fixed overnight in 1% paraformaldehyde (PFA; Thermo Scientific, JI9943.K2) at 4°C, then cryoprotected in a sucrose gradient (10%, 15%, and 30%, each for 16 h). Samples were embedded in Tissue-Tek OCT (Sakura Finetek 4583) on dry ice and sectioned using a Leica cryostat. Sections were washed twice with PBS (3 min each), permeabilized in PBST (0.5% Triton X-100 in PBS) for 15 min, and blocked for 1 h at room temperature in 10% BSA in PBST. Sections were incubated overnight at 4°C with primary antibodies diluted (Dystrophin antibody, Abcam, ab15277) in 5% BSA (Sigma, A7906-100G). After four PBST washes, slides were incubated for 2 h at room temperature with Hoechst 33342 (Sigma, B2261), species-specific Alexa Fluor 488 secondary antibodies (Thermo Fisher Scientific, A-11008) and a fluorescent probe complementary to the tcDNA sequence (5′-Alexa647– ATTCTGTGATCTTTCTTGTT-3′, Eurogentec). Samples were washed, mounted, and imaged using a Leica confocal microscope.

### Transcriptomic analysis

Total RNA was extracted from 3D cultures (day 12 of differentiation) using TRIzol reagent. Triplicate RNA samples (200 ng each) were processed by the NovaSeqX sequencing platform (Integragen, Evry, France; project PJ2311412). Libraries were prepared using the NEBNext Ultra II Directional RNA Library Prep Kit (Illumina) following the manufacturer’s recommendations. Poly(A)+ mRNA was purified using oligo(dT) magnetic beads (NEB), fragmented, reverse transcribed, and converted to double-stranded cDNA. After adapter ligation and PCR amplification, libraries were sequenced on an Illumina NovaSeqX instrument using paired-end 100 bp reads. Data processing was performed with Illumina Real-Time Analysis software. Bioinformatic analysis was conducted in Galiléo (Integragen), a cloud-based RNA-seq analysis platform. Quantification of gene expression were mapped with STAR to obtain the number of reads associated to each gene in the Gencode v.31 annotation (restricted to protein-coding genes, antisense, and lincRNAs). Raw counts for each sample were imported into R statistical software. Extracted count matrix was normalized for library size and coding length of genes to compute FPKM expression levels. The Bioconductor edgeR package was used for unsupervised analysis to import raw counts into R statistical software, and compute normalized log2 CPM (counts per millions of mapped reads) using the TMM (weighted trimmed mean of M-values) as normalization procedure. The normalized expression matrix from a custom gene list was used to classify the samples according to their gene expression patterns using PCA, hierarchical clustering and consensus clustering. PCA was performed by FactoMineR:PCA function with “ncp = 10, scale.unit = FALSE” parameters. Hierarchical clustering was performed by stats:hclust function (with Euclidean distance and ward.D method). Consensus clustering was performed by ConsensusClusterPlus function to examine the stability of the clusters. We established consensus partitions of the dataset in K clusters (for K = 2, 3, …, 8), on the basis of 1,000 resampling iterations (80% of genes, 80% of sample) of hierarchical clustering, with Euclidean distance and ward.D method. Then, the cumulative distribution functions (CDFs) of the consensus matrices were used to determine the optimal number of clusters (K = 3 for instance), considering both the shape of the functions and the area under the CDF curves. tSNE analysis was performed with the Bioconductor Rtsne package applied to the PCA object (theta = 0.0, perplexity = 1, max_iter = 1000). For differential expression analysis, the Bioconductor edgeR package was used to import raw counts into R statistical software. Differential expression analysis was performed using the Bioconductor limma package and the voom transformation. To improve the statistical power of the analysis, only genes expressed in at least one sample (FPKM ≥ 0.1) were considered. A qval threshold of ≤ 0.05 and a minimum fold change of 1.2 were used to define differentially expressed genes. Gene list from the differential analysis was ordered by decreasing log2 fold change. Pathway and gene set enrichment analysis was performed by clusterProfiler:GSEA function using the fgsea algorithm. Gene sets from MSigDB v666 database were selected among the H classes, keeping only gene sets defined by 666 genes. Thresholds for log_2_FC, FPKM, and FDR are detailed in the results section. All dataset is deposited in GEO under the accession number GSE317140.

### Proteomic analysis

Samples were lysed in RIPA buffer (Thermo Fisher Scientific, 89900), heated for 10 min at 95°C, and sonicated (Bioruptor; 10 cycles, 30 s on/off, 21°C). Four micrograms of protein were reduced and alkylated (5 mM TCEP, 15 mM CAA, 70°C, 10 min). Digestion followed the SP3 protocol^86^ using a 100:1 substrate:enzyme ratio. Magnetic beads were washed in 70% ethanol and acetonitrile, and proteins were digested overnight at room temperature with trypsin. Samples were acidified (0.1% formic acid) and desalted with SDB-RPS tips. Peptides were loaded onto EvoTips Pure and separated on an EvoSep One system (Evosep, Denmark) with a 15 cm PepSep column (150 μm ID, 1.5 μm particles) using a 30-sample-per-day method. Mobile phases consisted of 0.1% FA (A) and 0.1% FA in acetonitrile (B). The system was coupled to a timsTOF Pro 2 (Bruker) in dia-PASEF mode, calibrated with Agilent ESI-Low Tuning Mix. Proteomics data were deposited in the ProteomeXchange Consortium (PRIDE; dataset ID PXD060394).^87^ Files were processed with DIA-NN 1.9 in library-free mode against UniProt Human (2024). Mass deviation was set to 15 ppm. Downstream analyses were performed in R (v.4.2.2) using *diann*, *tidyverse*, *data.table*, *samr*, *vsn*, and *ggplot2*. Only unique peptides meeting quality thresholds (*q* < 0.01, ≥4 fragments, quantity quality >0.7) were retained. QC was assessed in Skyline-Daily (v.22.21.391). Further analysis was performed in Perseus (v.1.6.15.0) and InstantClue (v.0.10.10.20211105). MaxLFQ intensities were log_2_-transformed and filtered for 100% completeness in at least one group. ANOVA and unpaired Student’s *t* tests were applied with FDR <0.05 (500 randomizations). GO enrichment was performed on significantly altered proteins using *gProfiler* and *GOrilla*. All dataset is deposited in PRIME under the project accession number PXD060394.

### Statistical analysis

Statistical analyses were conducted using GraphPad Prism version 8 (GraphPad Software, San Diego, CA, USA). One- or two-way analyses of variance (ANOVA) were used to compare differences among multiple groups, followed by Dunnett or Tukey’s multiple comparisons test for post hoc pairwise analyses. Correlation analyses were assessed using Spearman’s rank correlation test. All data are presented as mean ± standard deviation (SD). *n* refers to the number of biological replicates per group. Statistical significance against respective negative control was defined as *p* < 0.05∗, *p* < 0.01∗∗, *p* < 0.001∗∗∗.

## Data and code availability

The datasets generated in the current study are available from the corresponding author upon reasonable request.

## Acknowledgments

This study was funded by the Association Monegasque contre les myopathies, Duchenne Parent Project France and SQY Therapeutics. We are grateful to Vincent Mouly (Institut de Myologie, Paris) for providing the immortalized myoblasts used in this study. We also thank Valérie Robain for her work on the APAFIS #34354–2021121417092550 v4 referral and Annemieke Aarstma-Rus as well as Johan T. den Dunnen (Leiden University Medical Center, the Netherlands) for providing the transgenic human DMD mice.

## Author contributions

S.G., M.G.-G., and L.G. designed the experiments. S.G., M.G.-G., C. Beley, T.H.N., S.C., and L.M.S. conducted the experiments. S.G., M.G.-G., C.B., T.H.N., S.C., L.M.S., C. Blaszykowski., M.K., and L.G. analyzed the data. S.G. and L.G. wrote the manuscript. All authors reviewed, edited and approved the final manuscript.

## Declaration of interests

L.G. is co-founder of SQY Therapeutics. S.G., M.G.G., C. Beley, T.H.N., S.C., and C. Blaszykowski were SQY Therapeutics employees at the time the work was conducted.

## Supplemental information

Document S1. Figures S1–S6 and Tables S1–S4

Document S2. Article plus supplemental information
