# Supplementary material for: Antisense oligonucleotide selection scheme for rare Duchenne muscular dystrophy mutations: Application to DMD exon 16 skipping
Source: Mol Ther Nucleic Acids. 2026 Aug 3;37(3):103048. doi: 10.1016/j.omtn.2026.103048 (PMC13520093; doi:10.1016/j.omtn.2026.103048)
Supplement: Supplementary file 1 [file mmc1.pdf]

OMTN, Volume *37*

Supplemental information

Antisense oligonucleotide selection scheme for rare Duchenne muscular dystrophy mutations: Application to *DMD* exon 16 skipping

Marine Geoffroy-Guiraud, Cyriaque Beley, Stephany Campuzano, Thu Huyen Nguyen, Luisa M. Schmidt, Christophe Blaszykowski, Marcus Krueger, Luis Garcia, and Simon Guiraud

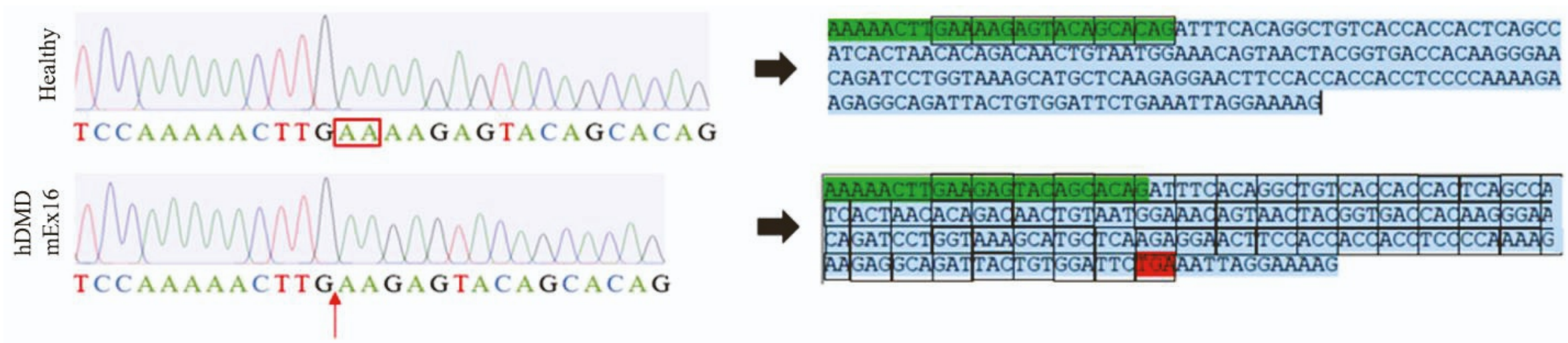

Figure S1. Sequencing validation of healthy and hDMD mEx16 cell lines. Sanger sequencing chromatograms showing a two-base pair deletion in the *hDMD mEx16* cell line, leading to a premature stop codon within exon 17 of the *dystrophin* gene sequence, compared to the healthy control.

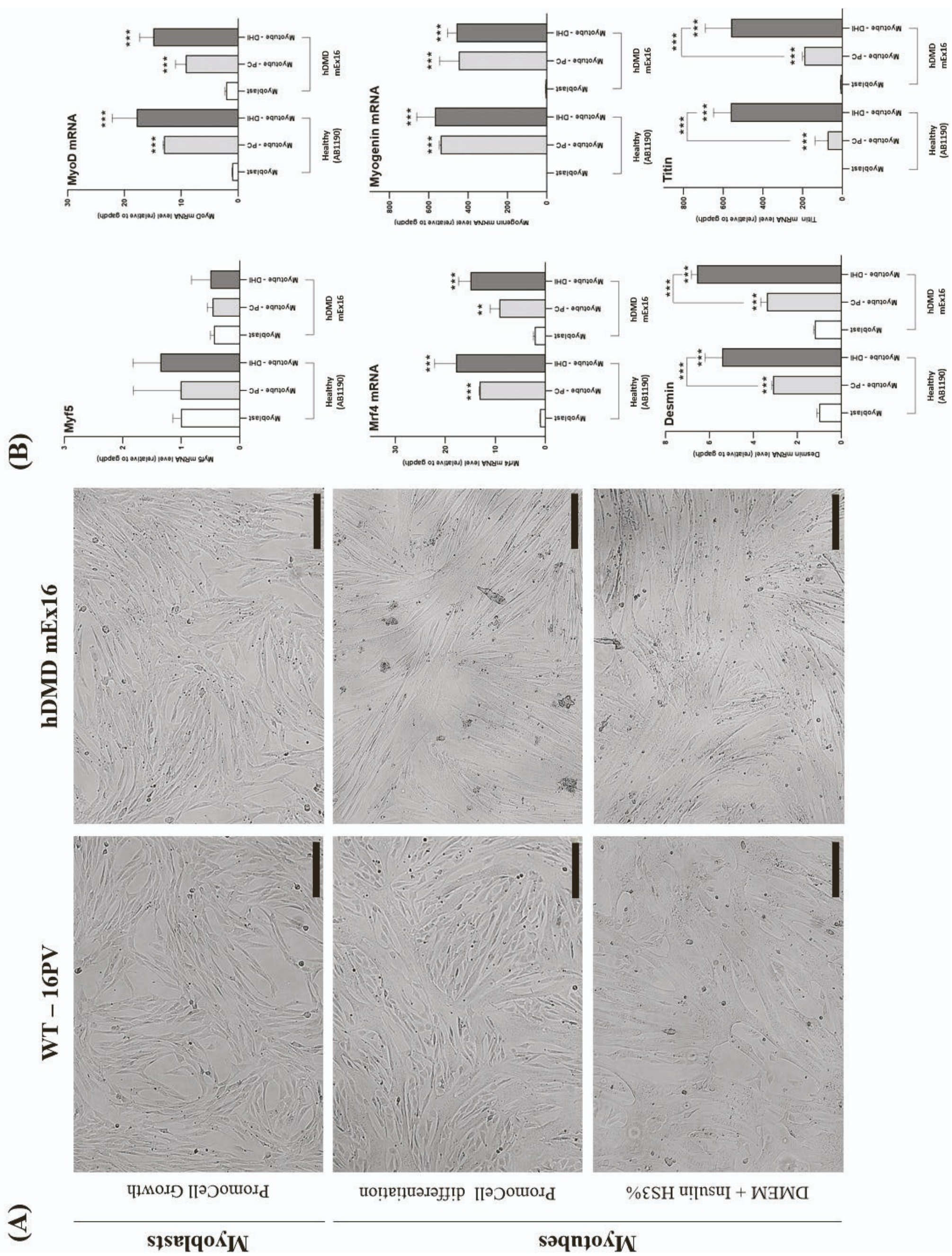

Figure S2. Myogenic potential of healthy and hDMD mEx16 cell lines. (A) Representative images of healthy and hDMD mEx16 myoblasts, and of differentiated myotubes obtained using either PromoCell differentiation medium or DMEM supplemented with insulin and 3% horse serum (DHI). Scale bar = 200 µm. **(B)** Relative gene expression of myogenic markers at different stages of differentiation: *Myf5* (myoblast marker), *MyoD* (myocyte marker), and *MRF4*, *myogenin*, *desmin*, and *titin* (myotube markers) in healthy and hDMD mEx16 myoblasts and myotubes. ($p < 0.05$ (*), $p < 0.01$ (**), $p < 0.001$ (***); analyzed by one-way ANOVA. Results are expressed as the mean ± SD (n = 3 per group)

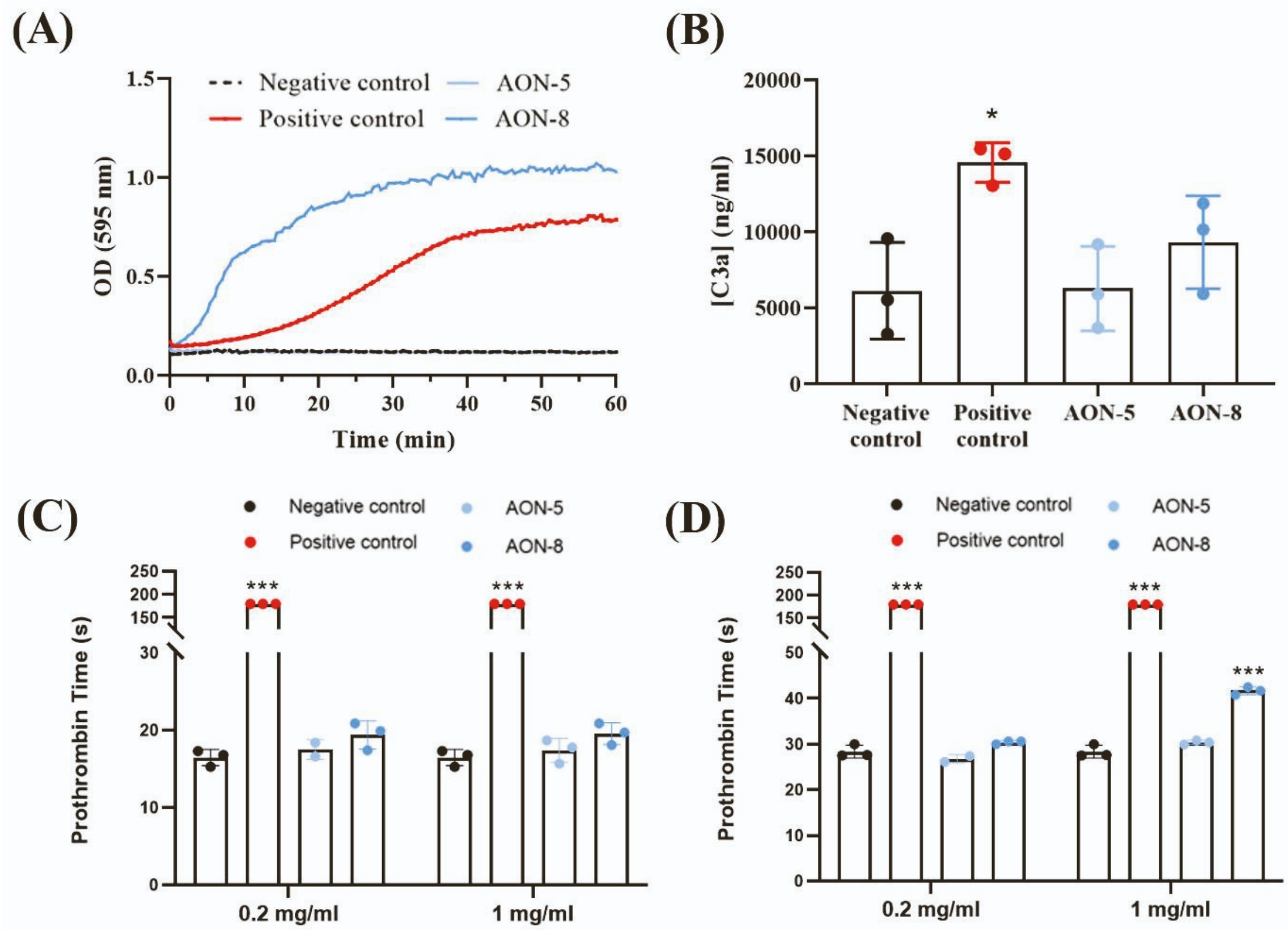

Figure S3. ***In vitro* toxicity assessment of exon 16–skipping tcDNA in NHP plasma. (A)** Flocculation assay performed with AON-5 and AON-8 in cynomolgus monkey plasma. **(B)** C3a complement activation assay in monkey serum following incubation with AON-5 and AON-8. **(C)** Prothrombin time (PT) and **(D)** activated partial thromboplastin time (APTT) assays evaluating the effects of AON-5 and AON-8 on coagulation parameters in monkey serum. ($p < 0.05$ (*), $p < 0.01$ (**), $p < 0.001$ (***); analyzed by one- or two-way ANOVA. Results are expressed as the mean ± SD (n = 3 per group).

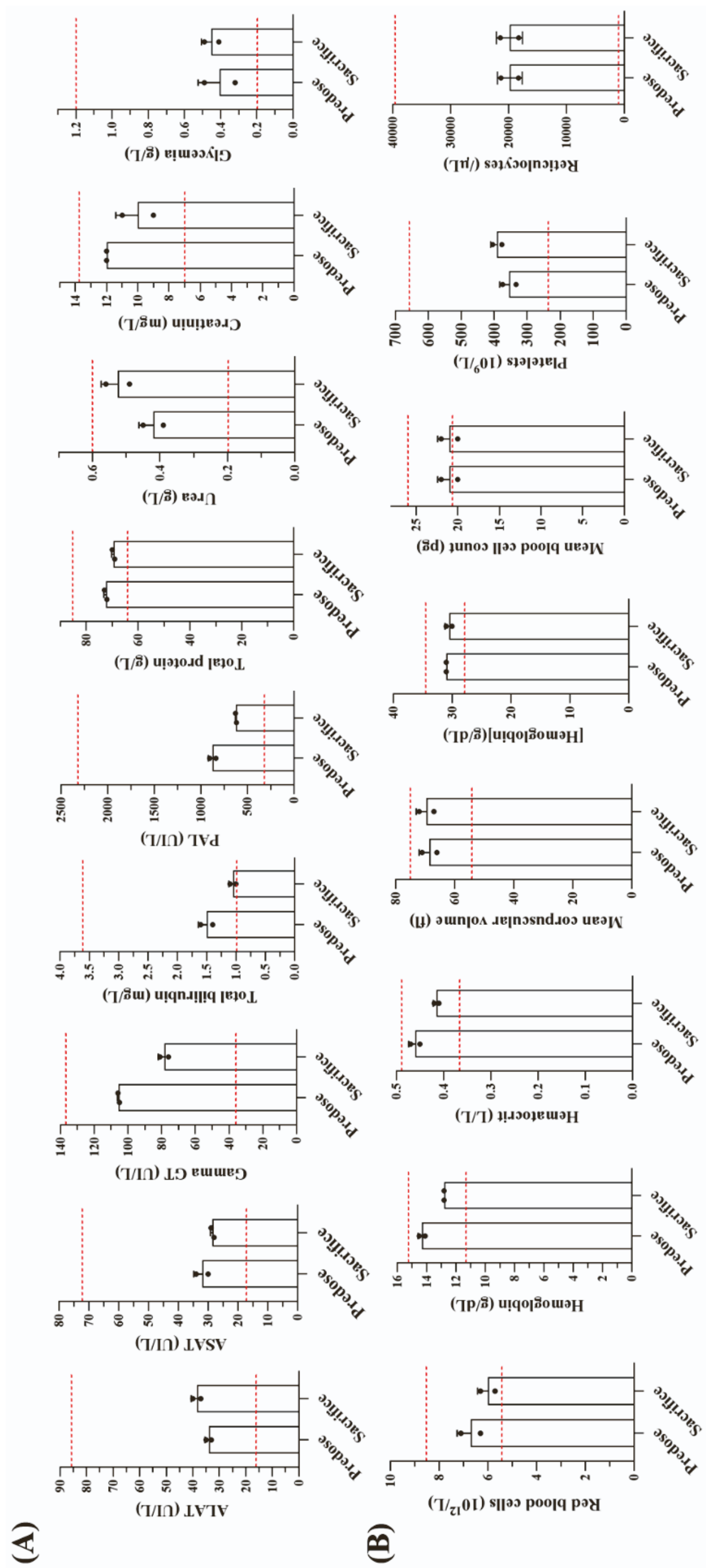

Figure S4. Liver and kidney safety biomarkers following PALM-SQY16 treatment in NHPs. (A) Serum levels of key biomarkers associated with liver and kidney function measured before and after a 4-week treatment with PALM-SQY16 (50 mg/kg/week). **(B)** Hematological profiling of multiple parameters to evaluate physiological or pathological changes potentially induced by PALM-SQY16 exposure. Red bars indicate the upper and lower limits of the normal NHP reference range. Results are presented as mean ± SD; n = 2 NHPs.

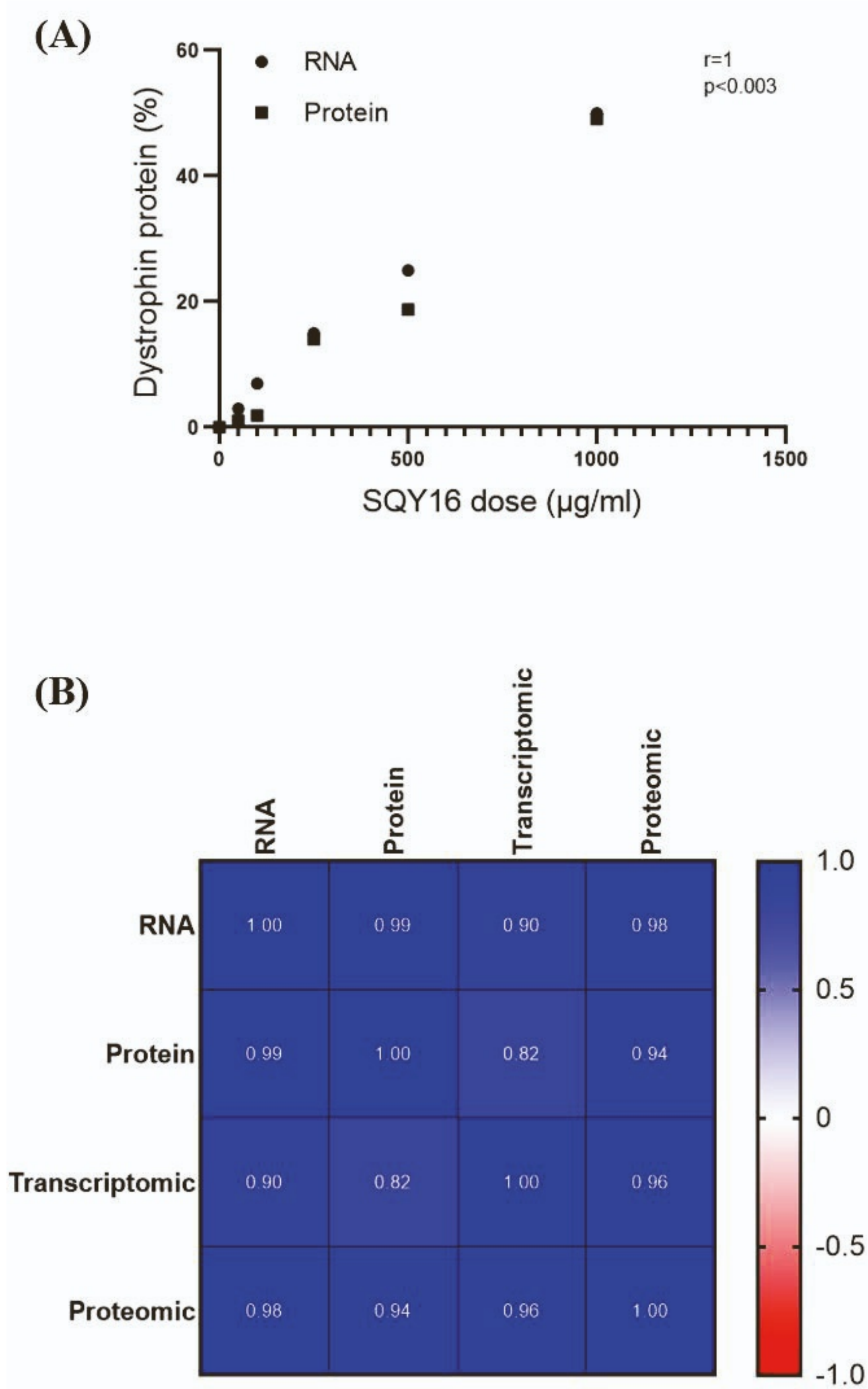

Figure S5. Correlation studies between exon-skipping, dystrophin restoration and transcriptomic and proteomic rescue (A) Spearman correlation analyses between exon-skipping level and dystrophin protein restoration in 3D mini-muscle study. **(B)** Multi-matrix Pearson analysis to correlate exon-skipping level and dystrophin protein restoration, transcriptomic and proteomic rescues in 3D muscle culture study.

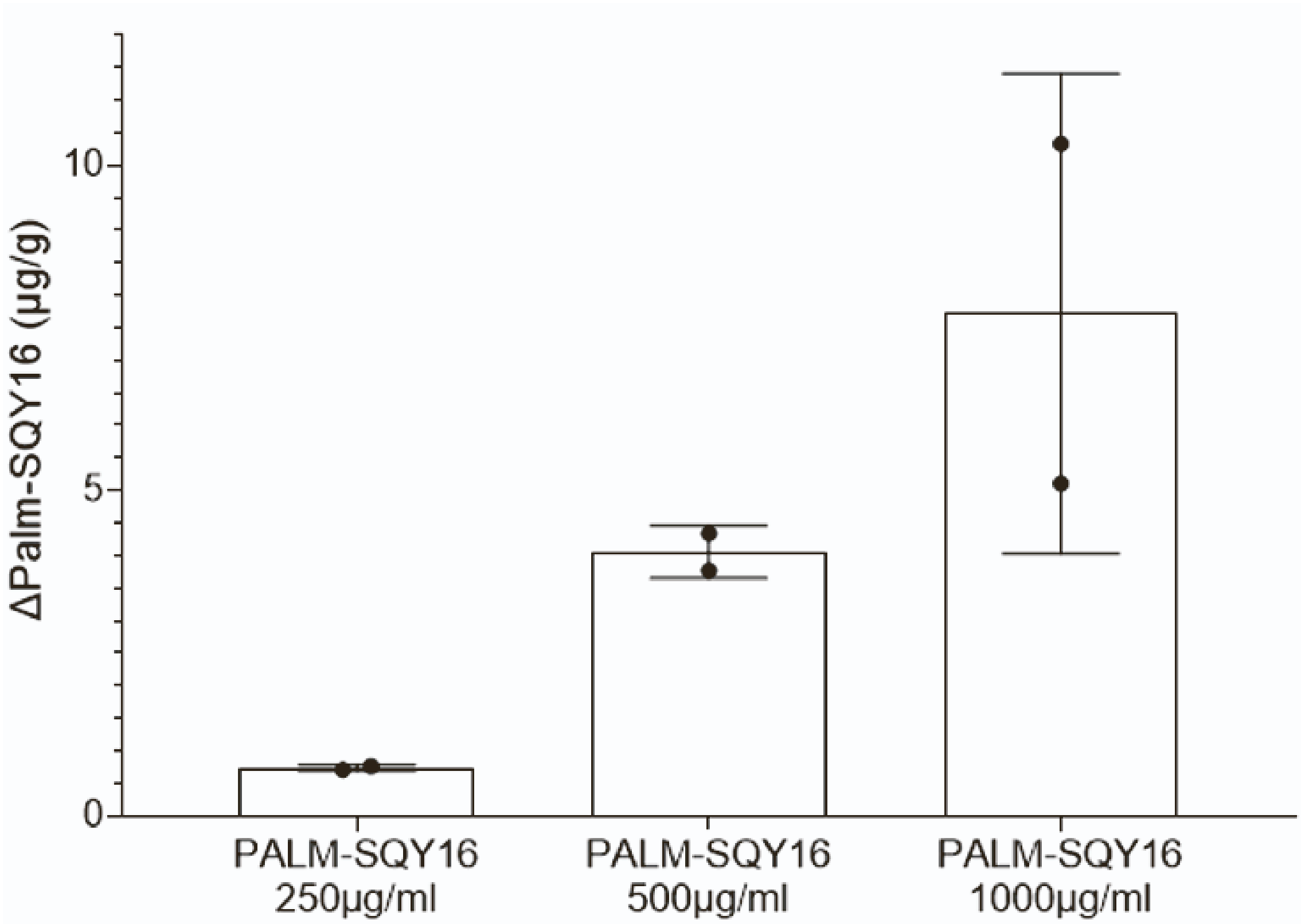

Figure S6. Quantification of the metabolite ΔPALM-SQY16 in hDMD 3D muscle cultures by automated capillary electrophoresis (QSEP100 system) after PALM-SQY16 treatment (250, 500 and 1000µg/ml every 48 hours with medium change for 10 days).

Table S1. List of tcDNA antisense oligonucleotides designed to induce skipping of DMD exon 16.

AON	Sequence (5'-3')	Localisation	Nucleotide count	GC%	Tm (°C)	Self-complementary
AON-1	ACTATAGTAONAAAONTGCTTT	Intron 15	15	30	50.2	No
AON-2	ACACCACTATAGTAON	Intron 15	15	40	40.6	No
AON-3	ATAONCATACACCACT	Intron 15	15	40	40.6	No
AON-4	CCTGGGTTGCATAONC	Intron 15	15	60	48.8	No
AON-5	AACAAGAAAGAAONACAGAAT	Intron 15	20	30	50.2	No
AON-6	CTGTTAAAACAAGAAAGAAON	Intron 15	20	30	50.2	No
AON-7	ACCTGTTAAAACAAGAAAGA	Intron 15	20	30	50.2	Yes
AON-8	CCGCTTTTAAAACCTGTTAA	Intron 15 - Exon 16 junction	20	35	52.3	Yes
AON-9	AONTAGAAONCGCTTTTAAAAC	Exon 16	20	35	52.3	No
AON-10	TTTAONTAGAAONCGCTTTTAA	Exon 16	20	30	50.2	No
AON-11	TAONTTTAONTAGAAONCGCTTT	Exon 16	20	35	52.3	No
AON-12	GTTTGCCCATGGATTGCTTT	Exon 16	20	45	56.4	No
AON-13	ACAGTTTGCCCATGGATTGC	Exon 16	20	50	58.4	No
AON-14	AATACAGTTTGCCCATGGAT	Exon 16	20	40	54.3	NO
AON-15	GTGAATACAGTTTGCCCATG	Exon 16	20	45	56.4	No
AON-16	TGAGTGAATACAGTTTGCCC	Exon 16	20	45	56.4	No
AON-17	GTTTGAGTGAATACAGTTTG	Exon 16	20	35	52.3	No
AON-18	CTTGTTTGAGTGAATACAGT	Exon 16	20	35	52.3	No
AON-19	GAAONTTGTTTGAGTGAATAC	Exon 16	20	35	52.3	No
AON-20	GAAGAAONTTGTTTGAGTGAA	Exon 16	20	35	52.4	No
AON-21	GTGTTGAAAGAAGAAONTTGT	Exon 16	20	35	52.3	No
AON-22	AONAGTGTTGAAAGAAGAAONT	Exon 16	20	35	52.3	No
AON-23	AONTAONAGTGTTGAAAGAAGA	Exon 16	20	35	52.3	Yes
AON-24	TATAONTAONAGTGTTGAAAGA	Exon 16	20	30	50.2	Yes
AON-25	ACTTATAONTAONAGTGTTGAA	Exon 16	20	30	50.2	No
AON-26	CTGACTTATAONTAONAGTGTT	Exon 16	20	35	52.3	No
AON-27	AONACTGACTTATAONTAONAGT	Exon 16	20	35	52.3	Yes
AON-28	GGGAONACTGACTTATAONTAON	Exon 16	20	45	56.4	No
AON-29	AONTGGGAONACTGACTTATAON	Exon 16	20	45	56.4	No
AON-30	AONTAONTGGGAONACTGACTTA	Exon 16	20	45	56.4	No
AON-31	CCGAONTAONTGGGAONACTGAC	Exon 16	20	60	62.5	No
AON-32	CTAONCGAONTAONTGGGAONACT	Exon 16	20	55	60.5	No
AON-33	ATGCTAONCGAONTAONTGGGAON	Exon 16	20	55	60.5	No
AON-34	GCCATGCTAONCGAONTAONTGG	Exon 16	20	60	62.5	No
AON-35	CCAGCCATGCTAONCGAONTAON	Exon 16	20	60	62.5	No
AON-36	TAAONCAGCCATGCTAONCGAON	Exon 16	20	55	60.5	No
AON-37	AGTTAAONCAGCCATGCTAONC	Exon 16	20	50	58.4	No
AON-38	CAAAGTTAAONCAGCCATGCT	Exon 16	20	45	56.4	No
AON-39	GGGCAAAGTTAAONCAGCCAT	Exon 16	20	50	58.4	No

AON-40	ACCGGGCAAAGTTAAONCAGC	Exon 16	20	55	60.5	No
AON-41	AACACCGGGCAAAGTTAAONC	Exon 16	20	50	58.4	No
AON-42	CCCAACACCGGGCAAAGTTA	Exon 16	20	55	60.5	No
AON-43	TAAONCCAACACCGGGCAAAG	Exon 16	20	55	60.5	No
AON-44	AATTAAONCCAACACCGGGCA	Exon 16	20	50	58.4	No
AON-45	CTAAATTAAONCCAACACCGG	Exon 16	20	45	56.4	No
AON-46	GGACTAAATTAAONCCAACAC	Exon 16	20	40	54.3	No
AON-47	TTTGGACTAAATTAAONCCAA	Exon 16	20	30	50.2	Yes
AON-48	GTTTTTGGACTAAATTAAONC	Exon 16	20	30	50.2	No
AON-49	CAAGTTTTTGGACTAAATTA	Exon 16	20	30	48.2	No
AON-50	TTAONAAGTTTTTGGACTAAA	Exon 16	20	30	48.2	No
AON-51	AONTTTAONAAGTTTTTGGACT	Exon 16	20	30	50.2	No
AON-52	TACAONTTTAONAAGTTTTTGG	Exon 16	20	30	50.2	No
AON-53	TAACCTATGCTGTACAONTTT	Exon 16 - Intron 17 junction	20	35	52.3	No
AON-54	CACTAACCTATGCTGTACAON	Exon 16 - Intron 17 junction	20	45	56.4	No
AON-55	TAAONACTAACCTATGCTGTA	Exon 16 - Intron 17 junction	20	35	52.3	No
AON-56	TGGTAAONACTAACCTATGCT	Exon 16 - Intron 17 junction	20	40	54.3	No
AON-57	AATTGGTAAONACTAACCTAT	Exon 16 - Intron 17 junction	20	30	50.2	No
AON-58	GATAATTGGTAAONACTAACC	Exon 16 - Intron 17 junction	20	35	52.3	No
AON-59	TGATAATTGGTAAONACTAAC	Exon 16 - Intron 17 junction	20	30	50.2	Yes

Table S2. Top 10 PALM-SQY16-binding serum proteins identified in human, NHP, and mouse samples.

Ranking	PG.Organisms	PG.ProteinDescriptions	Control	SQY16	SQY16 vs ctrl	PG.Pvalue
1	Homo sapiens	Albumin	4231.286133	110550	96	2.83E-10
1	Macaca fascicularis	Apolipoprotein A-I	7667.275391	135271	95	1.72E-08
1	Mus musculus	Albumin	1981.302979	41607.3	95	2.23E-12
2	Homo sapiens	Apolipoprotein A-I	10429.64844	71339.2	87	8.65E-10
2	Macaca fascicularis	Albumin	4467.617188	69193.1	94	1.26E-07
2	Mus musculus	Apolipoprotein A-II	2347.99292	31424.5	93	1.34E-11
3	Homo sapiens	Apolipoprotein B-100	8716.444336	56417.5	87	4.58E-11
3	Macaca fascicularis	Apolipoprotein C-I, basic form	4065.653076	52775.7	93	7.90E-08
3	Mus musculus	Apolipoprotein B	3075.102295	21329.6	87	2.72E-13
4	Homo sapiens	Serum amyloid A-4 protein	687.9754028	47228	99	2.95E-09
4	Macaca fascicularis	Vitellogenin domain-containing protein	3032.335449	40316.1	93	3.07E-08
4	Mus musculus	Albumin	1164.128418	17693.8	94	1.38E-10
5	Homo sapiens	Serum paraoxonase/arylesterase 1	3621.314697	43421.7	92	2.32E-09
5	Macaca fascicularis	Apolipoprotein A-II	5510.067383	38298.1	87	3.17E-07
5	Mus musculus	Apolipoprotein N	1109.661255	16573.4	94	4.56E-12
6	Homo sapiens	Zinc finger protein 263	0	33737.8	100	7.28E-05
6	Macaca fascicularis	Apolipoprotein C-II	4926.317383	27235.3	85	5.55E-07
6	Mus musculus	Serum paraoxonase/arylesterase 1	3610.830566	16024.9	82	1.32E-11
7	Homo sapiens	Apolipoprotein A-II	1819.417358	24869.8	93	2.03E-09
7	Macaca fascicularis	Zinc finger protein FPM315	0	20061.8	100	0.005710636
7	Mus musculus	C-X-C motif chemokine	1460.464233	10249.7	88	3.35E-12
8	Homo sapiens	Glycogen debranching enzyme	146.4835663	16482.2	99	4.68E-06
8	Macaca fascicularis	Lipid transfer protein II	1553.619141	19821.4	93	1.19E-07
8	Mus musculus	Metalloproteinase inhibitor 3	394.190155	7795.21	95	1.17E-11
9	Homo sapiens	Small integral membrane protein 18	0	13758.6	100	5.30E-06
9	Macaca fascicularis	Protein AMBP	369.6045227	16345.7	98	3.32E-06
9	Mus musculus	Apolipoprotein C-II	1215.479492	6298.31	84	1.82E-10
10	Homo sapiens	Apolipoprotein C-I	2101.688721	11304.9	84	1.45E-09
10	Macaca fascicularis	Apolipoprotein C-IV	993.6564941	9833.12	91	6.55E-06
10	Mus musculus	Fibrinogen-like 2	1074.525391	6137.43	85	1.12E-11

Table S3. Top 20 most significantly upregulated (A) and downregulated (B) genes in hDMD mEx16 3D muscle cultures compared to healthy.

(A) Top 20 most significantly upregulated genes									
Rank	Gene	Description	Gene position	Cumulative Length Exon	FPKM WT	FPKM DMD	log2FC	pval	qval
1	DDIT4	DNA damage inducible transcript 4	chr10:72273924-72276036	2054	78.44	213.13	1.56	2.59 E-15	2.16 E-12
2	ANKRD37	ankyrin repeat domain 37	chr4:185396021-185400628	2253	4.12	23.63	2.67	1.23 E-14	4.69 E-12
3	TMEM258	transmembrane protein 258	chr11:61768501-61792802	3068	12.96	38.29	1.69	2.55 E-14	6.82 E-12
4	VAMP8	vesicle associated membrane protein 8	chr2:85561562-85582031	895	32.98	93.48	1.63	2.64 E-14	6.92 E-12
5	CXCL10	C-X-C motif chemokine ligand 10	chr4:76021118-76023497	1175	5.93	60.23	3.55	3.64 E-14	8.08 E-12
6	CCL5	C-C motif chemokine ligand 5	chr17:35871491-35880793	1499	21.47	84.47	2.11	3.59 E-14	8.08 E-12
7	RPS24	ribosomal protein S24	chr10:78033760-78056813	7829	61.65	145.58	1.36	3.40 E-14	8.08 E-12
8	FAM162A	family with sequence similarity 162 member A	chr3:122384176-122412334	3793	32.46	68.91	1.21	3.69 E-14	8.08 E-12
9	RPS27L	ribosomal protein S27 like	chr15:63125872-63158021	8825	26.02	60.05	1.33	4.25 E-14	8.72 E-12
10	CTSL	cathepsin L	chr9:87726109-87731469	1852	68.84	182.89	1.54	4.69 E-14	9.24 E-12
11	AKR1B1	aldo-keto reductase family 1 member B	chr7:134442356-134459284	3654	126.14	307.33	1.41	5.68 E-14	1.04 E-11
12	ID2	inhibitor of DNA binding 2	chr2:8678845-8684461	2213	36.46	87.09	1.38	5.79 E-14	1.04 E-11
13	RPS5	ribosomal protein S5	chr19:58386400-58394806	3617	125.43	270.88	1.23	6.35 E-14	1.09 E-11
14	ATP5ME	ATP synthase membrane subunit e	chr4:672436-674330	932	55.24	138.59	1.45	7.53 E-14	1.21 E-11
15	RPL41	ribosomal protein L41	chr12:56116590-56117967	1108	397.51	967.98	1.41	7.44 E-14	1.21 E-11
16	RPS11	ribosomal protein S11	chr19:49496365-49499708	1409	499.29	1139.27	1.31	7.88 E-14	1.21 E-11
17	PLIN2	perilipin 2	chr9:19108375-19149290	5011	201.95	422.01	1.19	7.96 E-14	1.21 E-11
18	SSR4	signal sequence receptor subunit 4	chrX:153793516-153798505	4590	37.68	85.10	1.30	8.96 E-14	1.30 E-11
19	SNRPG	small nuclear ribonucleoprotein polypeptide G	chr2:70281362-70293740	1764	18.66	45.23	1.41	9.99 E-14	1.37 E-11
20	RPL35	ribosomal protein L35	chr9:124857880-124861981	1535	246.79	533.61	1.24	1.11 E-13	1.48 E-11
(B) Top 20 most significantly downregulated genes									
Rank	Gene	Description	Gene position	Cumulative Length Exon	FPKM WT	FPKM DMD	log2FC	pval	qval
1	COL6A3	collagen type VI alpha 3 chain	chr2:237324003-237414375	19633	187.01	4.06	-5.39	1.14 E-18	1.53 E-14
2	FN1	fibronectin 1	chr2:215360440-215436073	17216	1301.47	137.54	-3.12	1.40 E-16	8.50 E-13
3	CPE	carboxypeptidase E	chr4:165361194-165498547	3119	248.92	33.13	-2.78	1.91 E-16	8.50 E-13
4	TERT	telomerase reverse transcriptase	chr5:1253147-1295047	5899	274.3	32.34	-2.96	4.36 E-16	8.74 E-13

5	LRP1	LDL receptor related protein 1	chr12:57128493-57213351	20839	42.16	5.64	-2.77	4.10E-16	8.74E-13
6	AHNAK	AHNAK nucleoprotein	chr11:62433542-62556235	19466	41.7	6.83	-2.49	5.24E-16	8.74E-13
7	NEAT1	nuclear paraspeckle assembly transcript 1	chr11:65422774-65445540	22767	25.93	4.72	-2.33	4.76E-16	8.74E-13
8	IGFBP5	insulin like growth factor binding protein 5	chr2:216672105-216695549	6466	109.8	20.12	-2.32	3.02E-16	8.74E-13
9	TGFBI	transforming growth factor beta induced	chr5:136028988-136063818	7717	506.69	86.74	-2.42	7.16E-16	1.06E-12
10	C3	complement C3	chr19:6677704-6730562	8328	113.24	12.8	-3.02	9.80E-16	1.31E-12
11	HSPG2	heparan sulfate proteoglycan 2	chr1:21822244-21937310	18118	45.07	8.73	-2.24	1.08E-15	1.31E-12
12	LAMA2	laminin subunit alpha 2	chr6:128883141-129516569	10358	36.46	5.77	-2.53	1.21E-15	1.35E-12
13	CXCL6	C-X-C motif chemokine ligand 6	chr4:73836640-73849064	2040	203.93	16.48	-3.5	1.74E-15	1.79E-12
14	JAK2	Janus kinase 2	chr9:4984390-5128183	6170	32.55	2.88	-3.37	2.33E-15	2.16E-12
15	PADI2	peptidyl arginine deiminase 2	chr1:17066761-17119451	6000	30.12	8.13	-1.77	2.54E-15	2.16E-12
16	ZFP36L2	ZFP36 ring finger protein like 2	chr2:43222402-43226606	3693	54.13	8.1	-2.62	2.91E-15	2.28E-12
17	FKBP5	FKBP prolyl isomerase 5	chr6:35573585-35728583	10628	21.84	2.18	-3.2	3.33E-15	2.39E-12
18	FBN1	fibrillin 1	chr15:48408306-48645849	16057	23.62	3.74	-2.53	3.41E-15	2.39E-12
19	THBS1	thrombospondin 1	chr15:39581079-39599466	9158	149.05	38.32	-1.84	3.86E-15	2.46E-12
20	SERPING1	serpin family G member 1	chr11:57597387-57614853	3603	76	21.92	-1.67	3.87E-15	2.46E-12

Table S4. Top 20 most significantly upregulated (A) and downregulated (B) protein in hDMD mEx16 3D muscle cultures compared to healthy.

(A) Top 20 most significantly upregulated protein							
Rank	Gene	Description	Zscore WT	Zscore DMD	qval	-Log (pvalue DMD – WT)	Log2 Δ DMD-WT
1	CENPE	Centromere-associated protein E	-1.954615	0.510204667	0.005913	1.93133	2.47133
2	TAGLN	Transgelin	-1.883718667	0.420805	0.006991	1.85644	2.2937
3	MYH9	Myosin-9	-1.661322	0.421931667	0.013329	1.61432	2.28723
4	ACTG2	Actin, gamma-enteric smooth muscle	-1.603424333	1.329573333	0.001137	2.40998	2.1673
5	CSRP3	Cysteine and glycine-rich protein 3	-1.884676	0.606467333	0.007414	1.8116	2.16267
6	MICU2	Calcium uptake protein 2. mitochondrial	-1.928513333	0.593219667	0.00144	2.56071	2.14286
7	PDLIM3	PDZ and LIM domain protein 3	-1.772314	0.581498667	0.007692	1.81129	2.03867
8	ATP6V1G1	V-type proton ATPase subunit G 1	-1.99005	0.500961333	0.00225	2.86412	2.0227
9	PACSIN1	Protein kinase C and casein kinase substrate in neurons protein 1	-2.460325	0.370072333	0.001125	2.45006	2.01513
10	LDB3	LIM domain-binding protein 3	-1.822919	1.028777333	0.005964	2.08973	2.0057
11	PPIL1	Peptidyl-prolyl cis-trans isomerase-like 1	-1.993285	0.714247667	0.011582	1.69211	1.98542
12	SDHC	Succinate dehydrogenase cytochrome b560 subunit. Mitochondrial	-2.2673	0.584458667	0.010526	1.7582	1.97723
13	MYL9	Myosin regulatory light polypeptide 9	-1.829401	0.651729	0.011867	1.71523	1.9423
14	PYM1	Partner of Y14 and mago	-2.254095	0.612758033	0.006639	1.89964	1.86051
15	DPYSL3	Dihydropyrimidinase-related protein 3	-1.950863333	0.436403	0.007443	1.86409	1.83383
16	S100A4	Protein S100-A4	-1.94937	0.521460667	0.006169	2.09654	1.79173
17	COQ8A	Atypical kinase COQ8A. mitochondrial	-1.93316	0.210634467	0.003554	2.29549	1.75677
18	CAVIN3	Caveolae-associated protein 3	-2.44356	0.308698	0.006033	2.10548	1.75097
19	HRC	Sarcoplasmic reticulum histidine-rich calcium-binding protein	-1.809921333	0.512275103	0.007281	1.99882	1.7317
20	PACSIN3	Protein kinase C and casein kinase substrate in neurons protein 3	-1.863018	0.563883	0.011784	1.74703	1.72513
(B) Top 20 most significantly downregulated protein							
Rank	Gene	Description	Zscore WT	Zscore DMD	qval	-Log (pvalue DMD – WT)	Log2 Δ DMD-WT
1	SOD3	Extracellular superoxide dismutase [Cu-Zn]	2.041393333	-0.228013933	0	3.40916	-3.86227
2	FLG2	Filaggrin-2	1.577813333	-0.813425333	0	2.99483	-3.79477
3	FOLR1	Folate receptor alpha	1.498116667	-1.25099	0.002721	2.0903	-3.70422

4	HSPE1	10 kDa heat shock protein. mitochondrial	1.940356667	-0.755232333	0	2.8131	-3.54887
5	SPPL2A	Signal peptide peptidase-like 2A	2.283605	-0.295408667	0	3.37824	-3.34172
6	SOD2	Superoxide dismutase [Mn], mitochondrial	2.00912	-0.520433	0	3.67767	-3.20313
7	CALM1	Calmodulin-1	1.905606667	-0.658269667	0	3.46222	-3.1619
8	ICOSLG	ICOS ligand	1.95367	-0.6514509	0	2.96055	-3.16147
9	GLRX	Glutaredoxin-1	1.878566667	-0.750841	0.001964	2.57736	-3.09924
10	GSTT2	Glutathione S-transferase theta-2	2.021993333	-0.563733667	0	3.43551	-3.00143
11	CD36	Platelet glycoprotein 4	1.97422	-0.535542	0.001174	2.32432	-2.9249
12	CACNA2D1	Voltage-dependent calcium channel subunit alpha-2/delta-1	2.03965	-0.461582667	0	3.01587	-2.88837
13	CD81	CD81 antigen	1.957883333	-0.628808667	0.002118	2.66302	-2.835
14	JUP	Junction plakoglobin	1.789163333	-0.886918667	0.002699	2.14503	-2.82041
15	CNOT3	CCR4-NOT transcription complex subunit 3	2.516235	-0.399252667	0	3.51665	-2.74013
16	MYL4	Myosin light chain 4	1.981516667	-0.240078733	0.001459	2.47142	-2.64227
17	ARG1	Arginase-1	1.586905	-0.643713667	0.00108	2.31114	-2.63373
18	S100A7	Protein S100-A7	1.301635333	-0.474925333	0.001636	2.55784	-2.6182
19	CD47	Leukocyte surface antigen CD47	1.930926667	-0.749013	0	2.87836	-2.6141
20	SOD1	Superoxide dismutase [Cu-Zn]	1.916386667	-0.377113333	0.002842	2.81702	-2.60773
